# Translational readthrough of ciliopathy genes *BBS2* and *ALMS1* restores protein, ciliogenesis and function in patient fibroblasts

**DOI:** 10.1016/j.ebiom.2021.103515

**Published:** 2021-08-05

**Authors:** Jonathan Eintracht, Elizabeth Forsythe, Helen May-Simera, Mariya Moosajee

**Affiliations:** a UCL Institute of Ophthalmology, London, United Kingdom; b Clinical Genetics Unit, Great Ormond Street Hospital; c Genetics and Genomic Medicine Programme, Great Ormond Street Institute of Child Health; d Institute of Molecular Physiology, Johannes Gutenburg University, Mainz; e The Francis Crick Institute, London, United Kingdom; f Moorfields Eye Hospital NHS Foundation Trust, London, United Kingdom; g Great Ormond Street Hospital for Children NHS Foundation Trust, London, United Kingdom

**Keywords:** Nonsense suppression, Ciliopathies, Ataluren, Amlexanox, *BBS2*, *ALMS1*

## Abstract

**Background:**

Ciliary dysfunction underlies a range of genetic disorders collectively termed ciliopathies, for which there are no treatments available. Bardet-Biedl syndrome (BBS) is characterised by multisystemic involvement, including rod-cone dystrophy and renal abnormalities. Together with Alström syndrome (AS), they are known as the ‘obesity ciliopathies’ due to their common phenotype. Nonsense mutations are responsible for approximately 11% and 40% of BBS and AS cases, respectively. Translational readthrough inducing drugs (TRIDs) can restore full-length protein bypassing in-frame premature termination codons, and are a potential therapeutic approach for nonsense-mediated ciliopathies.

**Methods:**

Patient fibroblasts harbouring nonsense mutations from two different ciliopathies (Bardet-Biedl Syndrome and Alström Syndrome) were treated with PTC124 (ataluren) or amlexanox. Following treatment, gene expression, protein levels and ciliogenesis were evaluated. The expression of intraflagellar transport protein IFT88 and G-protein coupled receptor SSTR3 was investigated as a readout of ciliary function.

**Findings:**

mRNA expression was significantly increased in amlexanox-treated patient fibroblasts, and full-length BBS2 or ALMS1 protein expression was restored in PTC124- and amlexanox-treated fibroblasts. Treatment with TRIDs significantly improved ciliogenesis defects in BBS2^Y24*/R275*^ fibroblasts. Treatment recovered IFT88 expression and corrected SSTR3 mislocalisation in BBS2^Y24*/R275*^ and ALMS1^S1645*/S1645*^ fibroblasts, suggesting rescue of ciliary function.

**Interpretation:**

The recovery of full-length BBS2 and ALMS1 expression and correction of anatomical and functional ciliary defects in BBS2^Y24*/R275*^ and ALMS1^S1645*/S1645*^ fibroblasts suggest TRIDs are a potential therapeutic option for the treatment of nonsense-mediated ciliopathies.

**Research in context**

**Evidence before this study**

Ciliopathies are a diverse group of disorders caused by primary cilia dysfunction for which there are currently no disease-specific treatments available. Bardet-Biedl Syndrome (BBS) is the archetypal ciliopathy exhibiting multisystemic features associated with dysfunctional cilia. BBS shares many common phenotypes with another obesity-related ciliopathy, Alström Syndrome (AS). Nonsense mutations are responsible for approximately 11% and 40% of BBS and AS cases, respectively. These mutations introduce a premature termination codon (PTC) that halts translation. Translational readthrough inducing drugs (TRIDs) such as PTC124 (ataluren) and amlexanox can facilitate readthrough of PTCs to restore full-length protein expression. These have been effective in other *in vitro* models of choroideremia, Usher Syndrome and *RP2-*associated retinitis pigmentosa, but have yet to be tested in a human ciliopathy model.

**Added value of this study**

Our study is the first to report the efficacy of translational readthrough therapy in two distinct human ciliopathy models, namely in patient fibroblasts harbouring nonsense mutations in the *BBS2* and *ALMS1* genes*.* Following the treatment of patient cells with PTC124 or amlexanox we showed increased mRNA transcript levels and recovery of full-length BBS2 and ALMS1 protein expression.

Prior to treatment, we detected defective cilia formation in the *BBS2* patient fibroblasts and ciliary dysfunction in both cell lines. Following treatment, we were able to significantly improve cilia formation and recover function.

**Implications of all the available evidence**

Our study presents a novel therapy for the treatment of BBS and AS, for which no treatment exists. The recovery of full-length BBS2 and ALMS1 expression and correction of anatomical and functional ciliary defects suggests TRIDs are a potential therapeutic option for the treatment of nonsense-mediated ciliopathies. These results provide proof-of-concept for potential clinical translation and application to treat a wide spectrum of ciliopathies.

Alt-text: Unlabelled box

## 1 Introduction

Primary cilia are microtubule-based appendages that extend from the cell membrane. Not to be confused with motile cilia that move fluid across membrane surfaces, primary cilia are sensory organelles and regulate various signalling pathways such as sonic hedgehog (SHH), WNT, transforming growth factor-β (TGFβ) and others regulated by G-protein coupled receptors [1]. Ciliary dysfunction causes a range of disorders collectively termed ciliopathies [2]. Since primary cilia are found on virtually all vertebrate cell types, ciliary dysfunction can cause a multisystemic phenotype including retinopathy, obesity, polydactyly, cognitive impairment, renal abnormalities, central nervous system defects and craniofacial anomalies [2,3]. Non syndromic ciliopathies, where the ciliary dysfunction is confined to a single organ or tissue type, can also occur. Although ciliopathies are individually rare, collectively they are estimated to affect approximately 1:1000 people worldwide [4,5].

Ciliopathies are predominantly inherited in an autosomal recessive manner, however there are numerous reports implicating modifier alleles [6], [7], [8]. Most ciliopathy genes encode proteins related to ciliary trafficking or function. The ciliary axoneme is anchored to the cell via the basal body derived from the mother centriole [9], it is built and maintained via a specialised form of microtubule-based trafficking, termed intraflagellar transport (IFT) [10]. Precise coordination of ciliary trafficking and subsequent functional specificity is dependent not only on IFT, but also on regulatory aspects at the basal body.

Bardet-Biedl syndrome (BBS) is considered an archetypal ciliopathy, since patients exhibit the multisystemic features associated with ciliary dysfunction [11]. BBS is a valuable disease model for developing therapeutics that can be translated to other ciliopathies. Pathogenic variants in at least 23 genes (*BBS1-23),* with roles in cilia protein trafficking and IFT, have been identified and account for approximately 80% of BBS cases [11], [12], [13], [14]. No clear genotype-phenotype correlations exist, although some genes are associated with a specific phenotype, such as *BBS1* with milder renal involvement [15,16]. BBS1, BBS2, BBS4, BBS5, BBS7, BBS8, BBS9 and BBS18 comprise the BBSome, a highly conserved protein complex that plays a critical role in regulating cilia protein trafficking and IFT [17], [18], [19], [20], [21], [22]. Variants in *BBS2* (OMIM#606151) are responsible for 8-18% of BBS cases and cause a more severe retinal phenotype compared to *BBS1* [23], [24], [25], [26], [27]. The *BBS2* gene spans ~53 kb on chromosome 16q13, is comprised of 17 exons and encodes a 721-amino acid protein [27]. Of the 90 unique mutations documented in the Human Gene Mutation Database (HGMD, accessed April 2021), ~19% were pathogenic nonsense variants resulting in a premature termination codon (PTC). The most frequently detected variants are c.72C>G p.(Tyr24*), c.823C>*T p*.(Arg275*) and c.565C>*T, p*.(Arg189*) [26, 28].

In contrast to the locus heterogeneity associated with BBS, Alström syndrome (AS) is a monogenic ciliopathy associated exclusively with variants in *ALMS1* (OMIM#606844) [29]. Approximately 40% of pathogenic *ALMS1* variants are nonsense mutations, resulting in loss of function [29,30]. *ALMS1* spans ~230 kb on chromosome 2p13.1, and is comprised of 23 exons that encodes a 4169-amino acid protein [29]. ALMS1 is a basal body protein localised to the proximal end of the centriole and is therefore involved in ciliary protein transport and maintenance [29,[31], [32], [33]]. ALMS1 has a role in retinal development, guiding photoreceptor maturation and correct organisation, however its precise disease mechanism remains unclear [34], [35], [36], [37], [38].

There are currently no disease-specific treatments for any ciliopathy available to patients, and existing interventions are targeted towards symptomatic control such as obesity management [39,40]. Due to the systemic nature of BBS and AS, the current focus is to develop genetic therapies to correct the molecular defect globally [39]. Gene therapy would need to be delivered systemically, however, there are continued concerns regarding transfection of cells with adeno-associated viruses (AAV) or lentiviruses in terms of an induced cytotoxic immune response [10]. In addition, AAV vectors are limited by their cargo size (≤5 kb), making delivery of large genes such as many cilia proteins including *ALMS1* (cDNA 13 kb) difficult [41]. Furthermore, since ciliopathies display vast genetic heterogeneity, gene therapy will be limited due to cost. Thus other, more agnostic therapeutic options must be explored.

Translational readthrough or nonsense suppression therapy utilises TRIDs such as designer aminoglycosides (NB30, NB54, NB74, NB84, and NB124), and non-aminoglycoside small molecule drugs including PTC124 (3- [5-(2-Fluorophenyl)-1,2,4-oxadiazol-3-yl] benzoic acid or ataluren), readthrough compounds (RTC13 and RTC14), amlexanox (2-amino-7-isopropyl-5-oxo-5H-chromeno[2,3-b]pyridine-3-carboxylic acid) and DAP (2,6 – diaminopurine) [42], [43], [44], [45], [46], [47], [48]. Traditional aminoglycosides such as geneticin and G418 are most efficacious yet have high cytotoxicity [49]. The exact mechanism of action for most TRIDs is unknown, however, aminoglycosides are known to bind to the ribosomal A-site, disrupting its recognition of PTCs and permitting the insertion of a near-cognate tRNA instead of the eukaryotic release factors [50]. This facilitates readthrough of the PTC and translation of a full-length functional protein reaching up to 60% of wildtype levels in some studies [50,51].

PTC124 (Ataluren) treatment of select non-syndromic and syndromic retinal dystrophies has been previously described, restoring significant function in choroideremia (CHM) patient dermal fibroblasts, human induced pluripotent stem cell (iPSC) derived retinal pigment epithelium (RPE) and the *chm^ru848^* transgenic zebrafish, in *RP2-*related retinitis pigmentosa (RP) hiPSC-RPE, and in cell culture models and organotypic retinal cultures from *USH2A* and *USH1C-*associated Usher syndrome patients [43,45,52,53]. In 2014, PTC124 was approved as a treatment for Duchenne Muscular Dystrophy by the European Medicines Agency. Amlexanox has traditionally been used for the treatment of asthma and mouth ulcers but through a luciferase-based screening system, it was identified as a powerful dual-purpose drug that could inhibit nonsense-mediated mRNA decay and maintain translational readthrough properties [54]. Readthrough efficiency may be improved by increasing the number of stable mRNA transcripts available through NMD inhibition [54].

This is the first study to demonstrate a successful treatment for BBS and AS ciliopathies with nonsense suppression therapy. We treated BBS2^Y24*/R275*^ and ALMS1^S1645*/^^S1645*^ patient fibroblasts with PTC124 and amlexanox, as both compounds have previously been used in patients. Increased restoration of transcript and protein expression was accompanied by improvement of ciliogenesis and ciliary function defects, highlighting the possible utility of TRIDs to treat a range of nonsense mutation-dependent ciliopathies.

## 2 Methods

### 2.1 Ethics

The study protocol adhered to the tenets of the Declaration of Helsinki and received ethical approval from the National Research Ethics Service West Midlands Committee - Staffordshire, REC reference 11/WM/0127 for skin biopsies as part of the EURO-Wabb (European Wolfram, Alstrom and Bardet-Biedl syndrome) study. Written, informed consent was obtained from all participants prior to their inclusion in this study.

### 2.2 Study subjects and clinical features

Skin biopsies were obtained from a healthy control (WT), a *BBS2* patient with molecularly confirmed c.72C>*G, p*.(Tyr24*) in exon 1 (UAG stop codon) and c.823C>*T, p*.(Arg275*) in exon 8 (UAA stop codon), and an *ALMS1* patient with molecularly confirmed c.4937C>*A, p*.(Ser1645*) and c.4937C>*A, p*.(Ser1645*) in exon 8 (both UAA stop codons).

The BBS2^Y24*/R275*^ patient was a 17-year-old male, who presented with typical BBS criteria including rod-cone dystrophy, obesity, renal dysplasia, polydactyly and a typical facial gestalt. Additional characteristic features included speech delay, strabismus and a high arched palate. The patient was diagnosed at 2 years of age and followed up annually by a specialist BBS team. He was registered severely sight impaired at the age of 16. This patient is referred to as BBS2-P1 in this study. All mutations are in reference to *BBS2* canonical transcript ENST00000245157.5.

The ALMS1^S1645*/^^S1645*^ patient, referred to as P14 from a previous study [55], was an 18-year-old male who displayed nystagmus and photophobia by 3 months of age and was registered severely sight impaired by the age of 13. The patient also has bilateral sensorineural hearing loss, supported by hearing aids, obesity, diabetes mellitus and fatty liver disease with elevated plasma triglyceride and low HDL cholesterol indicative of metabolic dyslipidaemia. There were no observed cardiac or renal anomalies at time of biopsy. All mutations are in reference to *ALMS1* canonical transcript ENST00000613296.4.

The wild type control fibroblasts, who has not appeared in any other study, were derived from a 28-year old healthy Caucasian male with no known medical history or mutations in any known ciliopathy genes.

### 2.3 Fibroblast cell culture and dosing

Four-millimetre punch skin biopsies were taken under local anaesthetic from a healthy control, BBS2^Y24*/R275*^and ALMS1^S1645*/S1645*^ patients and processed as previously described [49]. Fibroblasts were maintained in fibroblast growth media (DMEM (ThermoFisher Scientific, cat#41966029), 15% FBS (ThermoFisher Scientific, cat#10500064), 1% P/S (ThermoFisher Scientific, cat#15140122), that was replaced every 4–5 days and cells passaged at 80-90% confluency. Prior to plating, fibroblasts were serum starved to promote ciliation (DMEM, 1% P/S) for 24 h. Fibroblasts were plated at 300,000 cells per well in a 12-well plate or 600,000 per well in a 6-well plate and serum-starved for a further 24h. Cells were subsequently dosed with 40 µM PTC124 (SelleckChem, TX, USA, cat#S6003) or 250 µM amlexanox (Abcam, UK, ab142825, cat#ab142825) as previously described [49,56].

### 2.4 Western blotting

Samples were analysed by western blotting as previously described [49]. Briefly, cells were washed with ice-cold PBS and total protein extract was prepared with RIPA buffer (ThermoFisher Scientific, MA, USA, cat#89900) at a ratio of 5 × 10^6^ cells/mL buffer and 1x Halt ™ protease and phosphatase inhibitor cocktail (ThermoFisher Scientific, MA, USA, cat#78440). Proteins were resolved on 4–15% Mini-PROTEAN® TGX ™ gels (Bio-RadInc., CA, USA, cat#4561025) and transferred to an Immun-Blot™ PVDF membrane using a Trans-Blot® SD semi-dry transfer cell (Bio-Rad Inc., CA, USA). Membranes were blocked with 5% non-fat dry milk in 0.1% PBS/T for 1 h. A complete list of primary and secondary antibodies used can be found in the supplementary information. Blots were scanned using the ChemiDoc XRS ™ Imaging System (Bio-Rad Inc., CA, USA) and quantitatively analysed using the Fiji/ImageJ software (National Institutes of Health, MD, USA, RRID: SCR_002285).

### 2.5 Immunocytochemistry

Cells were collected 24h post-dosing and washed in PBS before fixing in ice-cold methanol, permeabilised in PBS-0.3% Triton X-100 and consequently blocked with 10% Normal Goat Serum (ThermoFisher Scientific, MA, USA, cat#31872). Cells were incubated overnight at 4 °C with primary antibodies found in supplementary Table 1. Alexa-Fluor® 488- or 647-conjugated secondary antibodies were used for primary antibody detection. Slides were mounted using ProLong^TM^ Diamond Antifade Mountant with DAPI (ThermoFisher Scientific, MA, USA, cat#P36962). Imaging was performed using the Zeiss LSM 710 fluorescence microscope (Zeiss Research, Germany) and images were processed using Fiji/Image J. **Supplementary**

### 2.6 RT-qPCR

RT-qPCR was performed as previously described [57]. Briefly, RNA extraction was performed using the RNeasy Mini Kit (QIAGEN, Germany, cat#Z4014) and cDNA was synthesized from 500 ng using the SuperScript III First Strand cDNA synthesis kit (Invitrogen, CA, USA, cat#18080051) according to the manufacturer's instructions. Transcript levels were analysed using SYBR Green MasterMix (ThermoFisher Scientific, MA, USA, cat#4309155) on a StepOne Real-Time PCR system (Applied Biosystems, ThermoFisher, Paisley, UK). All transcript levels were measured in triplicate. Primers used for the RT-qPCR are listed in supplementary Table 2. *GAPDH, ACTB* and *G6PD* were used as reference genes and transcript levels were normalised to WT undosed fibroblasts.

### 2.7 Statistical analysis

Statistical analysis was performed using Excel (Microsoft, WA, USA). Where relevant, the normality of the data was initially confirmed using Q-Q plot analysis and the Shapiro-Wilk test. A two-tailed unpaired Student's t-test was used for comparison studies. A *p*-value of <0.05 was considered statistically significant. Significance levels were set when *P* < 0.05 (*), *P* < 0.01 (**), *P* < 0.001 (***). All results are expressed as mean ± SEM, unless specified. All experiments were performed with *n* = 3 biological replicates.

### 2.8 Role of the funding source

The funder of the study had no role in study design, data collection, data analysis, data interpretation or writing of the report. The corresponding authors had full access to all the data in the study and accept responsibility to submit for publication.

## 3 Results

### 3.1 Treatment of BBS2^Y24*/R275*^ fibroblasts with TRIDs restored full-length BBS2 expression

**Fig. 1:**
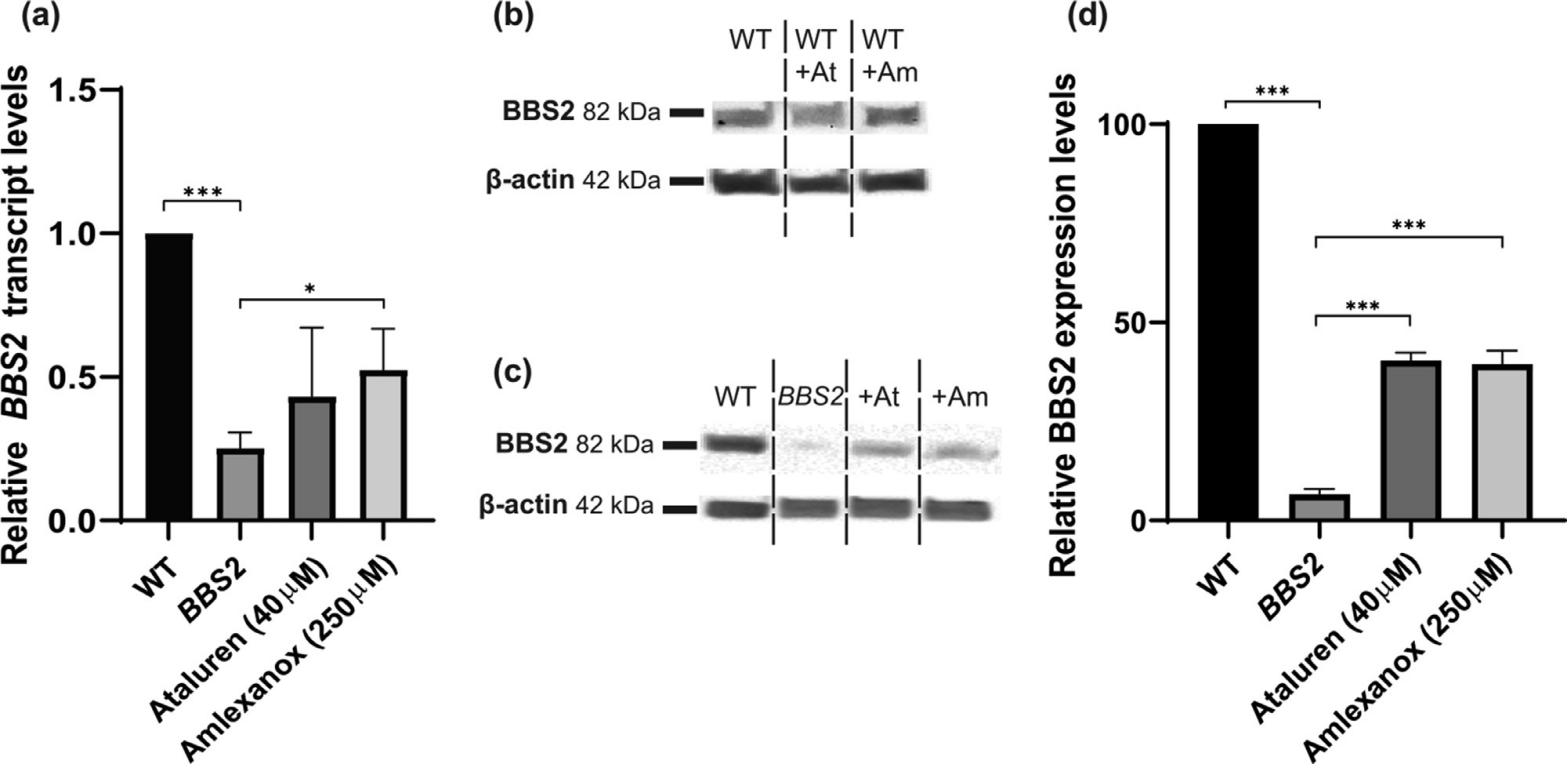
Restoration of BBS2 expression in treated BBS2^Y24*/R275*^ fibroblasts. (a) Relative expression levels of *BBS2* in wild type (WT), *BBS2* (untreated) and PTC124- and amlexanox-treated BBS2^Y24*/R275*^ fibroblasts by RT-qPCR. The levels of *BBS2* mRNA were normalised to *GAPDH, ACTB* and *G6PD* mRNA levels and untreated, PTC124- and amlexanox-treated levels normalised to WT. Experiments were performed in triplicate. (b) Western blot analysis of BBS2 expression in WT cells following treatment with PTC124(+At) or amlexanox (+Am) by western blot. β-actin was used as a loading control. (c) Western blot analysis of BBS2 expression in WT, *BBS2* (untreated), PTC124- (+At) and amlexanox- (+Am) treated BBS2^Y24*/R275*^ fibroblasts. β-actin was used as a loading control. (d) Representative relative band intensity of *BBS2*, PTC124- and amlexanox-treated BBS2^Y24*/R275*^ fibroblasts. BBS2 expression was normalised to β-actin levels and quantified as a percentage of WT BBS2 expression. (*P* < 0.05 (*), *P* < 0.01 (**), *P* < 0.001 (***) determined by Student's t-test in (a) and (d). Values are mean ± SEM, *n* = 3.

We assessed the efficacy of PTC124 and amlexanox treatment to (i) inhibit nonsense-mediated decay (NMD) and (ii) readthrough nonsense mutations and restore full-length functional BBS2 in a BBS2^Y24*/R275*^ patient dermal fibroblast line. RT-qPCR was performed to assay the levels of *BBS2* mRNA transcript in wild-type (WT), untreated BBS2^Y24*/R275*^ and *BBS2*^Y24*/R275*^ fibroblasts treated with either 40µM PTC124 or 250 µM amlexanox in three independent experiments. Untreated BBS2^Y24*/R275*^patient cells revealed significantly reduced *BBS2* transcripts reaching 20% of WT levels (*P* < 0.0001, Student's t-test). However, treatment with either PTC124 or amlexanox significantly increased *BBS2* transcripts to 43.23 ± 23.9% and 52.4 ± 14.37% (*P* < 0.013, Student's *t*-test) of WT levels respectively, but did not differ significantly between drugs (*P* < 0.497, Student's t-test) (Fig. 1a).

Western blot was performed to detect BBS2 protein expression in untreated and treated WT fibroblasts (with either 40 µM PTC124 or 250 µM amlexanox) as controls, however no significant difference was seen (*P* < 0.64 and *P* < 0.17 respectively, Student's *t*-test) (Fig. 1b). BBS2 expression was barely detectable in untreated BBS2^Y24*/R275*^ fibroblasts (Fig. 1c). However, following treatment of patient fibroblasts, BBS2 expression was restored to 40.40 ± 2.03% and 39.41 ± 3.46% of WT levels with 40 µM PTC124 and 250 µM amlexanox, respectively (Fig. 1c-d). These data suggest that both PTC124 and amlexanox can inhibit NMD to similar degrees and influence translational readthrough in BBS2^Y24*/R275*^ fibroblasts, restoring equal amounts of full-length BBS2 protein. In order to assess restored BBS2 protein functionality, we examined the effects of TRID treatment on the correction of anatomical and functional cilia defects.

### 3.2 Treatment of BBS2^Y24*/R275*^ fibroblasts with TRIDs significantly improved ciliogenesis

**Fig. 2:**
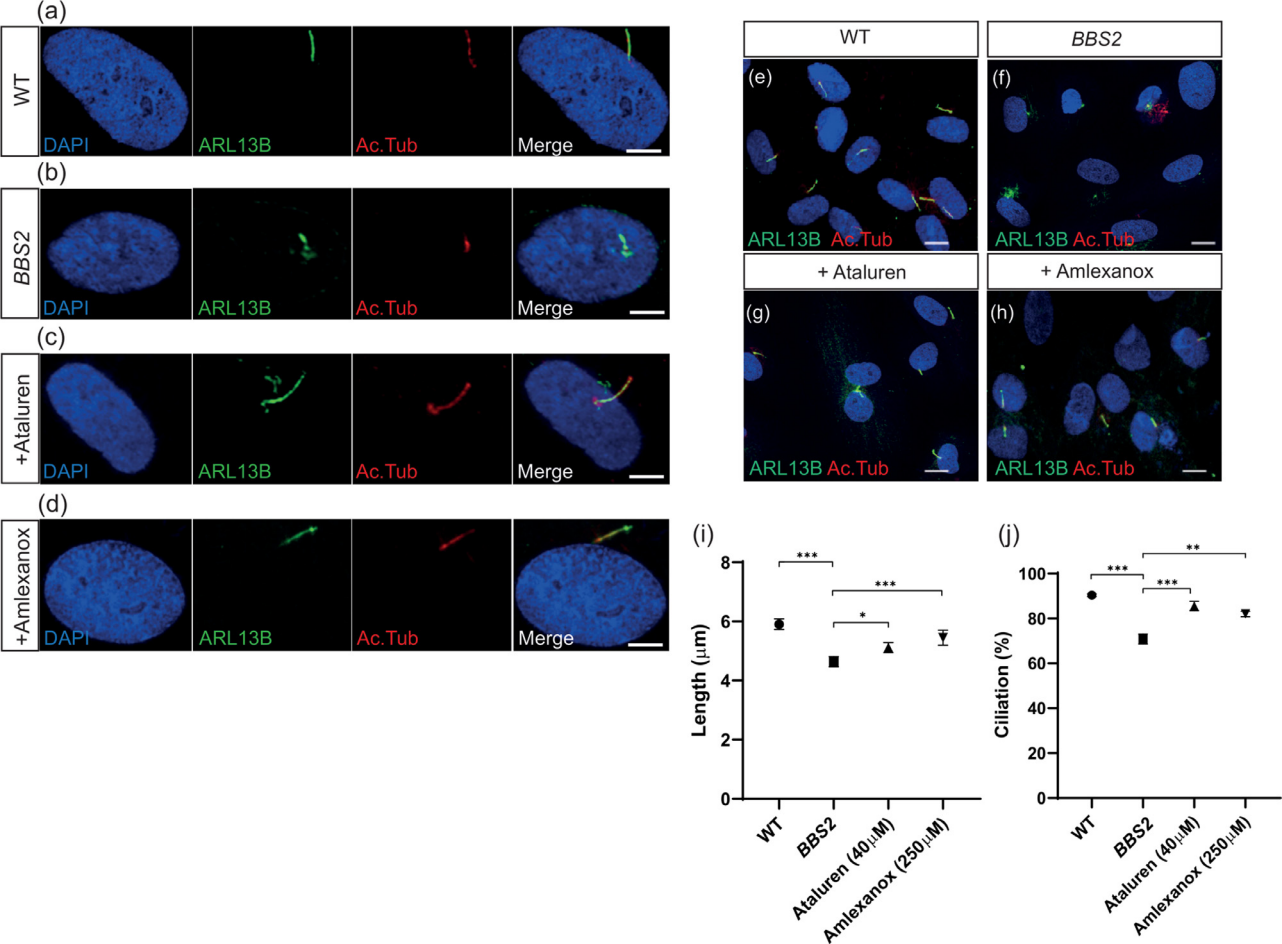
Ciliogenesis in BBS2^Y24*/R275*^ fibroblasts improved to wild type-like levels following treatment with PTC124 or amlexanox. (a-d) Immunofluorescent staining of individual (a) wild type (WT), (b) *BBS2* (untreated) (c) PTC124-treated BBS2^Y24*/R275*^ and (d) amlexanox-treated BBS2^Y24*/R275*^ fibroblasts. The cilium was detected by anti-ARL13B (shown in green) and anti-acetylated tubulin (shown in red) co-localization. (e-h) Representative immunofluorescent staining of cilia in WT, *BBS2* and PTC124- and amlexanox-treated BBS2^Y24*/R275*^ fibroblasts as detected using anti-ARL13b (shown in green) and anti-acetylated tubulin (shown in red) co-localisation. (i) Average cilia length measured in WT fibroblasts (*n* = 103), *BBS2* (*n* = 103), PTC124-treated (*n* = 104) and amlexanox-treated (*n* = 108) BBS2^Y24*/R275*^ fibroblasts. (j) Ciliation proportions in WT fibroblasts (*n* = 103), *BBS2* (*n* = 103), PTC124-treated (*n* = 104) and amlexanox-treated (*n* = 108) BBS2^Y24*/R275*^ fibroblasts. Cilia detected by ARL13B and acetylated tubulin co-localisation were counted. (*P* < 0.05 (*), *P* < 0.01 (**), *P* < 0.001 (***) determined by Student's t-test.

Since the BBSome is required for primary cilia formation and signalling function, the downstream effects of the rescue of BBS2 protein expression in patient fibroblasts were investigated via restoration of ciliogenesis (cilia length and proportion of ciliated cells (ciliation)) upon treatment with PTC124 or amlexanox (Fig. 2a-d). The ciliary membrane marker ARL13B and acetylated tubulin were used to label the microtubule axoneme in all conditions (*n* = 3, with minimum 100 cells analysed per replicate per condition) (Fig. 2a-d). Quantification of ciliary length revealed that average cilia length was significantly reduced by 22% from 5.93 ± 0.18 µm in WT fibroblasts to 4.64 ± 0.17µm in untreated BBS2^Y24*/R275*^ fibroblasts (*P* < 0.0001, Student's *t*-test) (Fig. 2e, 2f and 2i). Treatment with PTC124 significantly increased average cilia length by 10% to 5.11 ± 0.16 µm (*P* < 0.049, Student's *t*-test), although this was still significantly shorter than WT levels (*P* < 0.001, Student's *t*-test) (Fig 2e-i). Treatment with amlexanox was more effective as it significantly increased cilia length by 17% in amlexanox-treated cells to 5.45 ± 0.25 µm (*P* < 0.009, Student's *t*-test), which did not differ significantly from WT levels (*P* < 0.146, Student's t-test) (Fig. 2e-i). Ciliation was also significantly reduced in mutant BBS2^Y24*/R275*^ fibroblasts with 70.87 ± 1.21% (*n* = 73/103) cells ciliated, compared to 90.29 ± 3.23% (*n* = 93/103) in WT fibroblasts (*P* < 0.0001, Student's *t*-test) (Fig. 2j). Treatment with PTC124 or amlexanox both significantly increased the proportion of ciliated cells to 85.57 ± 1.62% (*n* = 89/104) (*P* < 0.00029, Student's *t*-test) and 82.41 ± 1.47% (*n* = 89/108) (*P* < 0.0087, Student's *t*-test) respectively (Fig. 2j). This suggests that treatment with PTC124 and amlexanox can rescue defective ciliation following restoration of full-length BBS2 protein.

### 3.3 Treatment with TRIDs significantly restored cilia function in BBS2^Y24*/R275*^ fibroblasts

**Fig. 3:**
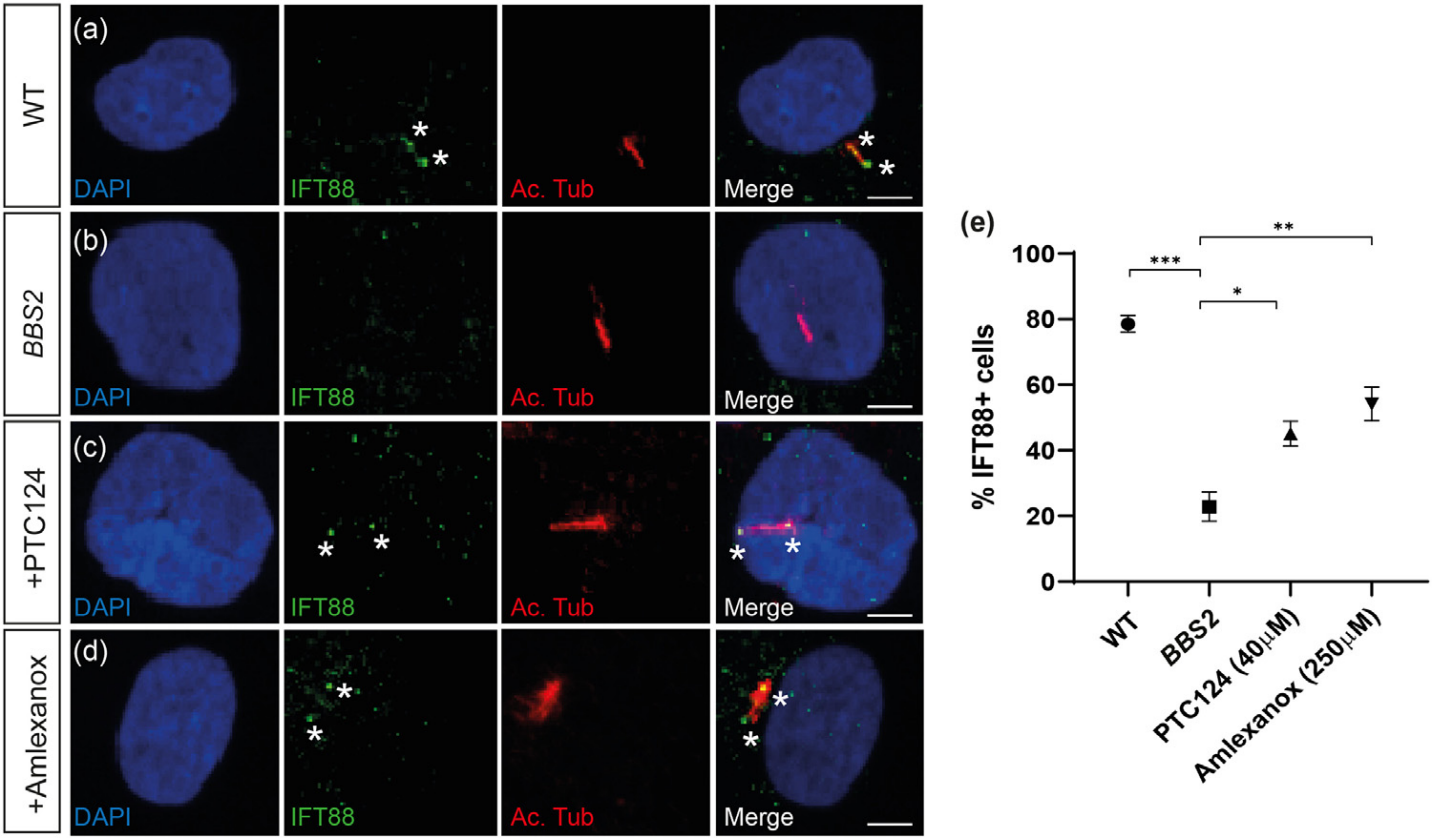
IFT88 expression is restored in BBS2^Y24*/R275*^ fibroblasts treated with TRIDs. (a-d) Representative immunofluorescent staining of cilia in (a) wild type (WT), (b) *BBS2* (untreated)*,* (c) PTC124- and (d) amlexanox-treated BBS2^Y24*/R275*^ fibroblasts shown by anti-acetylated tubulin (shown in red). IFT is detected as anti-IFT88 expression (shown in green) along the ciliary axoneme possibly accumulating at the tip. (e) The proportion of IFT88+ cells in WT fibroblasts (*n* = 94), *BBS2* (*n* = 102), PTC124-treated (*n* = 101) and amlexanox-treated (*n* = 98) BBS2^Y24*/R275*^ fibroblasts. *P* < 0.05 (*), *P* < 0.01 (**), *P* < 0.001 (***). Scale bar: 10µm. * indicates IFT88 expression.

BBS2 is a critical component of the BBSome, which co-operatively binds the IFT-A and IFT-B subunits to form the IFT complex that facilitates ciliary protein trafficking. IFT88 is a critical component of the IFT-B subunit and the ciliary transport machinery, its ciliary localisation is indicative of functional IFT along the cilium. IFT88 expression was detected in 78.72 ± 2.57% of WT cells (*n* = 74/94) along the ciliary axoneme with accumulation at the ciliary tip (Fig. 3a and 3e). IFT88 expression was significantly reduced in BBS2^Y24*/R275*^ patient cilia, with only 22.54 ± 4.52% of cells (*n* = 23/102) displaying any ciliary localisation (*P* < 0.00094, Student's t-test) (Fig. 3b and 3e). Following treatment with both PTC124 and amlexanox, IFT88 expression was restored in 45.54 ± 3.80% (*n* = 46/101) (*P* < 0.0048, Student's *t*-test) and 54.08 ± 5.19% (*n* = 53/98) (*P* < 0.0085, Student's *t*-test) of BBS2^Y24*/R275*^ patient cilia, respectively (Fig. 3c-3e).

**Fig. 4:**
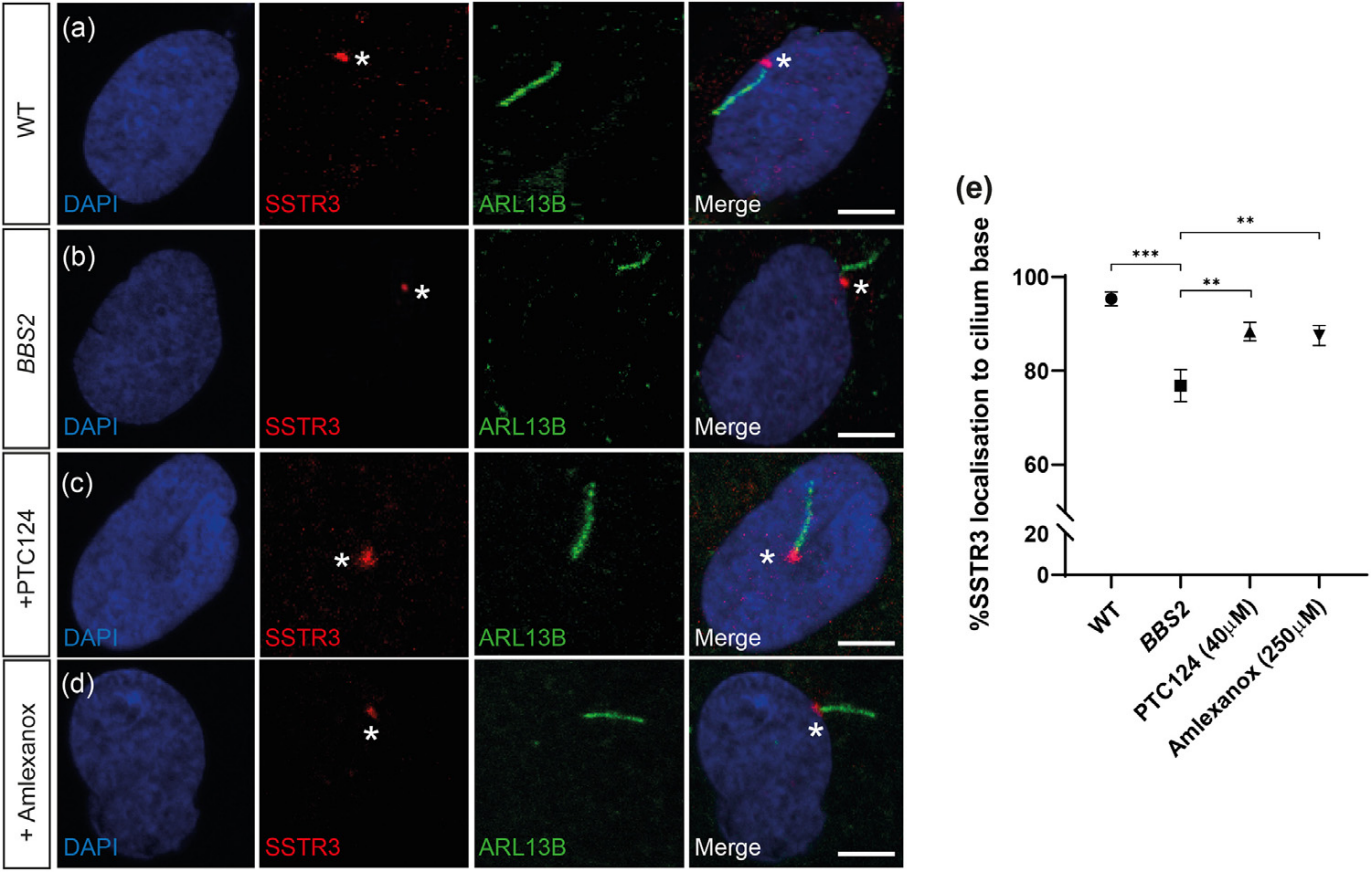
SSTR3 localisation is restored in BBS2^Y24*/R275*^ fibroblasts following treatment with TRIDs. (a-d) Representative immunofluorescent staining of cilia in (a) wild type (WT), (b) *BBS2* (untreated), (c) PTC124- and (d) amlexanox-treated BBS2^Y24*/R275*^ fibroblasts shown by anti-ARL13B (shown in green) staining the ciliary membrane and anti-SSTR3 (shown in red) staining adjacent to the ciliary membrane. (e) The percentage of correct SSTR3 localisation adjacent to the ciliary membrane in WT (*n* = 108), *BBS2* (*n* = 104), PTC124-treated (*n* = 104) and amlexanox-treated (*n* = 105) BBS2^Y24*/R275*^ fibroblasts. *P* < 0.05 (*), *P* < 0.01 (**), *P* < 0.001 (***). Scale bar: 10µm. * indicates SSTR3 expression.

The G-protein coupled receptor SSTR3 was used as an indicator of correct ciliary trafficking of protein cargo. Although it was not detected along the ciliary axoneme in WT fibroblasts, possibly as a consequence of cell-specific entry into the cilium, a clear localisation of SSTR3 was detected at the base of the cilium directly connecting to the ciliary axoneme in 95.37 ± 1.49% cells (*n* = 103/108) (Fig. 4a). In BBS2^Y24*/R275*^ fibroblasts, SSTR3 was significantly mislocalised away from the cilium and only directly attached to the axoneme in 77.89 ± 3.78% of cells (*n* = 81/104) (*P* < 0.002, Student's t-test) (Fig. 4b). Treatment of cells with PTC124 and amlexanox restored SSTR3 localisation adjacent to the axoneme, significantly increasing SSTR3 localisation in 91.09 ± 1.62% (*n* = 92/101) (*P* < 0.0014) and 90.48 ± 2.43% (*n* = 95/105) (*P* < 0.0057, Student's *t*-test) of cells, respectively (Fig. 4c-e). These data suggest a significant rescue of ciliary function along with ciliogenesis in patient cells following TRID treatment.

### 3.4 Treatment of ALMS1^S1645*/S1645*^ fibroblasts with TRIDs restored full-length ALMS1 expression to the basal body of the cilium

**Fig. 5:**
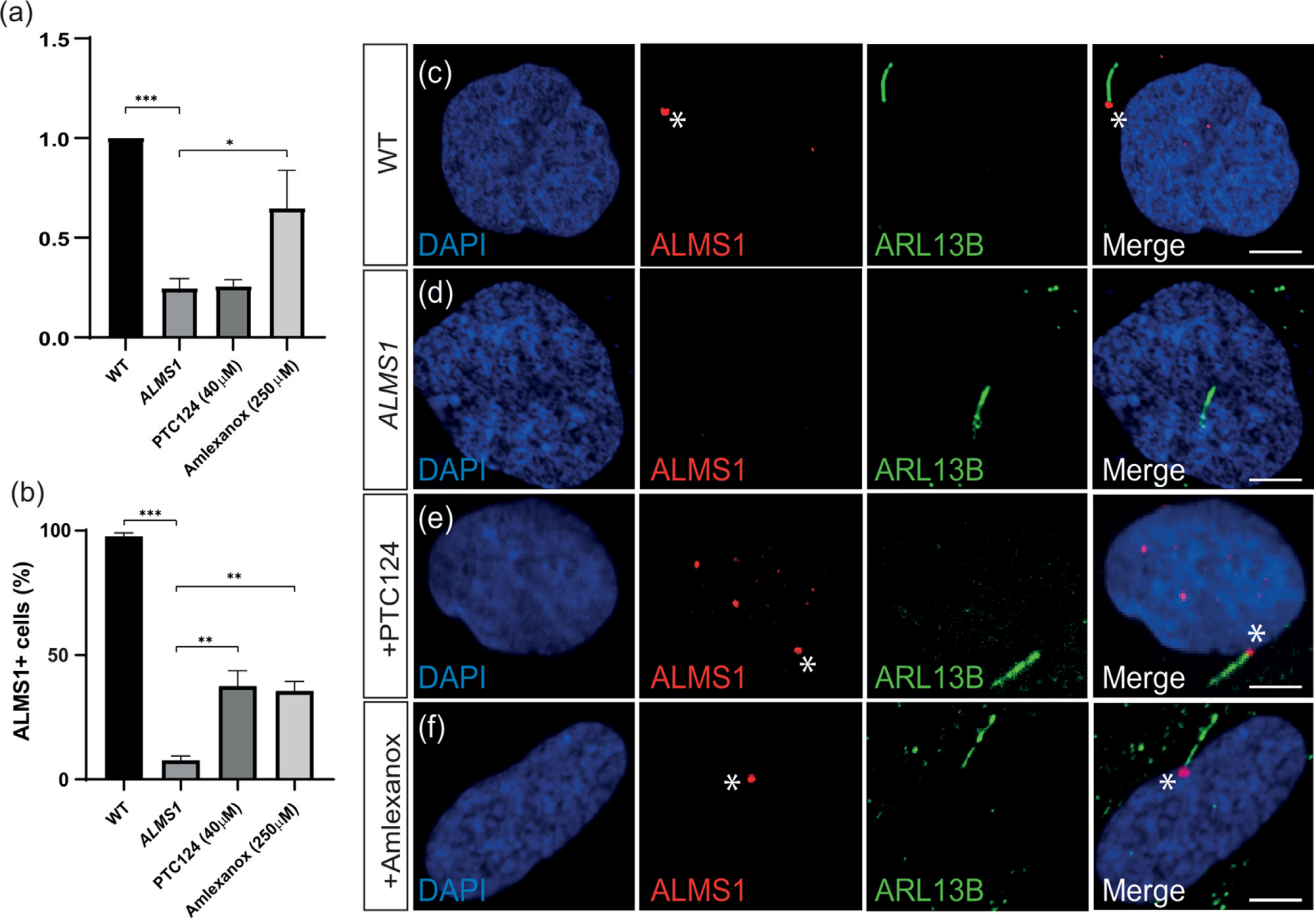
Restoration of ALMS1 expression in treated ALMS1^S1645*/S1645*^ fibroblasts. (a) Relative expression of *ALMS1* in wild type (WT), *ALMS1* (untreated) and PTC124- and amlexanox-treated ALMS1^S1645*/S1645*^ fibroblasts by RT-qPCR. The levels of *ALMS1* mRNA were normalised to *GAPDH, ACTB* and *G6PD* mRNA and untreated, PTC124- and amlexanox-treated levels normalised to WT levels. Experiments were performed in triplicate. (b) Quantification of ALMS1+ cells in WT (*n* = 112), *ALMS1* (*n* = 114) and PTC124- (*n* = 101) and amlexanox-treated (*n* = 102) ALMS1^S1645*/S1645*^ fibroblasts. (c-f) Representative immunofluorescent staining of cilia in (c) WT, (d) *ALMS1*, (e) PTC124- and (f) amlexanox-treated ALMS1^S1645*/S1645*^ fibroblasts shown by anti-ARL13B (shown in green) staining the ciliary membrane and anti-ALMS1 (shown in red) staining in the basal body complex. *P* < 0.05 (*), *P* < 0.01 (**), *P* < 0.001 (***). Scale bar: 10 µm. * indicates ALMS1 expression. Values are mean±SEM, *n* = 3.

Primary dermal fibroblasts from an Alström syndrome patient (ALMS1^S1645*/S1645*^) were used as a second ciliopathy model to assess the efficacy of PTC124 and amlexanox. RT-qPCR from ALMS1^S1645*/S1645*^ fibroblasts found that *ALMS1* mRNA transcripts were significantly reduced to 24.65 ± 6.91% of WT levels. Following treatment with amlexanox, this increased significantly to 64.82 ± 19.03% of wild type levels (*P* < 0.013, Student's t-test), similar to the effect observed in BBS2^Y24*/R275*^ cells (Fig. 5a). Treatment with PTC124 did not significantly alter *ALMS1* transcript levels compared to untreated ALMS1^S1645*/S1645*^ fibroblasts, reaching only 25.71 ± 3.28% of wild type levels (*P* < 0•41, Student's t-test) (Fig. 5a). Immunostaining of WT fibroblasts with an antibody against ALMS1 found protein expression localised to the base of the cilia adjacent to the ciliary axoneme in 97.32 ± 1.34% (*n* = 109/112) of healthy cells. This was significantly reduced in ALMS1^S1645*/S1645*^ fibroblasts, where ALMS1 was only present in 7.67 ± 1.76% (*n* = 9/114) of cilia (*P* < 0.0001, Student's *t*-test) (Fig. 5b-d). Treatment of ALMS1^S1645*/S1645*^ fibroblasts with TRIDs significantly restored ALMS1 protein localisation in 37.50 ± 6.23% of PTC124-treated (*n* = 38/101) (*P* < 0.0031, Student's *t*-test) and 35.29 ± 3.86% (*n* = 36/102) (*P* < 0.00046, Student's t-test) of amlexanox-treated cells (Fig. 5b and 5e-f).

### 3.5 Ciliogenesis was not impaired in ALMS1^S1645*/S1645*^ fibroblasts

**Fig. 6:**
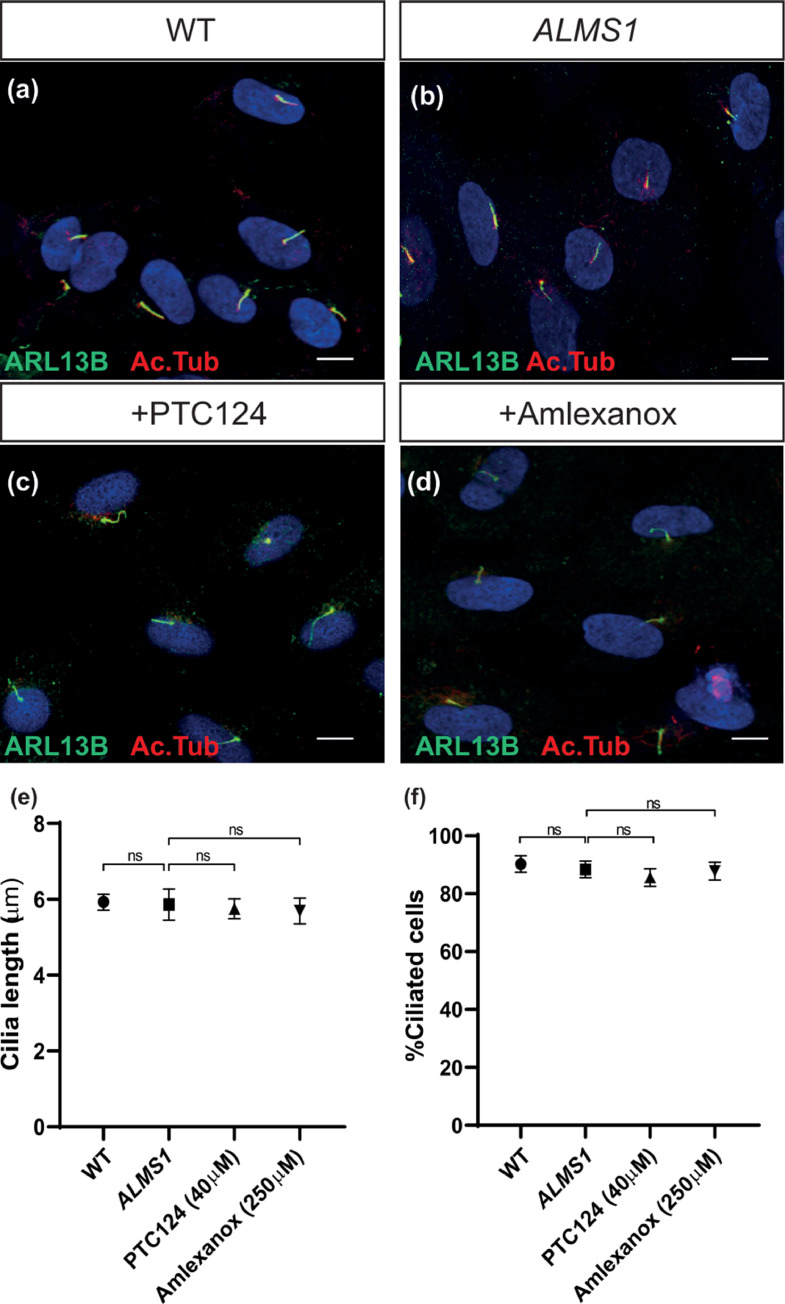
Ciliogenesis of PTC124 and amlexanox treated ALMS1^S1645*/S1645*^ fibroblasts. (a-d) Representative immunofluorescent staining of cilia in (a) wild type (WT), (b) *ALMS1* (untreated), (c) PTC124- and (d) amlexanox-treated *ALMS1*^S1645*/S1645*^ fibroblasts as detected using anti-ARL13b (shown in green) and anti-acetylated tubulin (shown in red) co-localisation. (e) Average cilia length measured in WT fibroblasts (*n* = 103), *ALMS1* (*n* = 102), PTC124-treated (*n* = 101) and amlexanox-treated (*n* = 99) ALMS1^S1645*/S1645*^ fibroblasts. (f) Ciliation proportions in wild type fibroblasts (*n* = 103), *ALMS1* (*n* = 102), PTC124-treated (*n* = 101) and amlexanox-treated (*n* = 99) ALMS1^S1645*/S1645*^ fibroblasts. Cilia determined by ARL13B and acetylated tubulin co-localisation were counted. *P* < 0.05 (*), *P* < 0.01 (**), *P* < 0.001 (***) determined by Student's t-test. Values are mean±SEM.

Ciliogenesis as measured by ARL13B and acetylated tubulin colocalization was not impaired in ALMS1^S1645*/S1645*^ fibroblasts, with no significant anatomical defects regarding cilia length and ciliation observed (*n* = 3, with minimum 100 cells analysed per replicate per condition) (Fig. 6a-f). Cilia length measured 5.86 ± 0.27 µm in ALMS1^S1645*/S1645*^ fibroblasts compared to 5.93 ± 0.18 µm in WT (*P* < 0.84, Student's *t*-test) (Fig. 6e). No change to cilia length was observed in patient fibroblasts treated with PTC124 or amlexanox with mean lengths of 5.75 ± 0.19 µm (*P* < 0.52, Student's *t*-test) and 5.69 ± 0.21 µm respectively (*P* < 0.67, Student's *t*-test) (Fig. 6e).

Similar levels of ciliation were observed between WT and ALMS1^S1645*/S1645*^ fibroblasts at 90.29 ± 3.23% (*n* = 93/103) and 88.23 ± 2.06% (*n* = 90/102) respectively (*P* < 0.61, Student's t-test) (Fig. 6f). Additionally, *ALMS1*^S1645*/S1645*^ fibroblasts did not significantly differ following treatment with PTC124 and amlexanox with ciliation levels of 85.14 ± 3.36% (*n* = 86/101) (*P* < 0.44, Student's *t*-test) and 87.88 ± 2.57% (*n* = 87/99) (*P* < 0.95, Student's *t*-test), respectively (Fig. 6f).

### 3.6 Restoration of cilia function in ALMS1^S1645*/S1645*^ fibroblasts

**Fig. 7:**
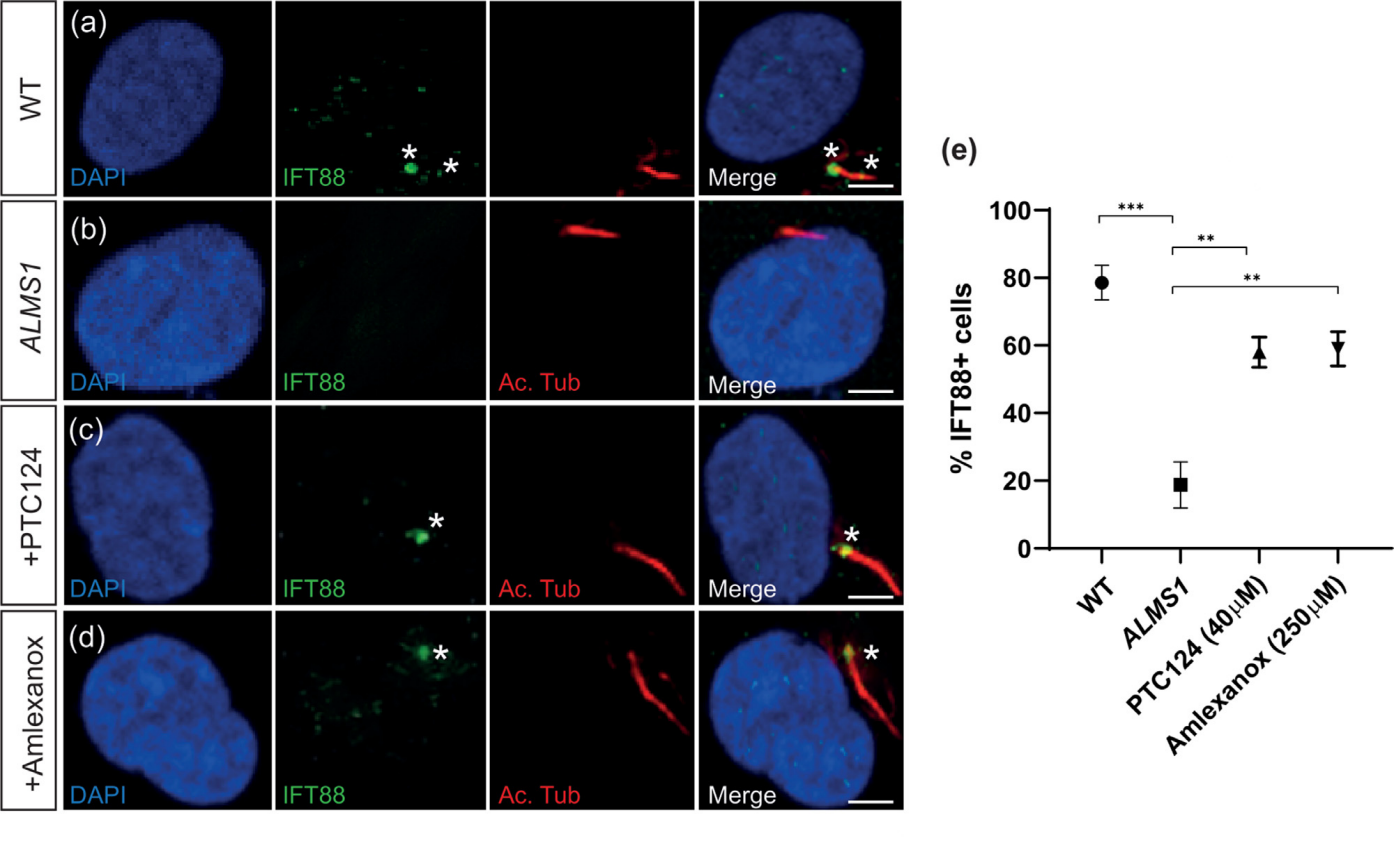
IFT88 expression is restored in ALMS1^S1645*/S1645*^ fibroblasts treated with TRIDs. (a-d) Representative immunofluorescent staining of cilia in (a) wild type (WT), (b) *ALMS1* (untreated), (c) PTC124- and (d) amlexanox-treated ALMS1^S1645*/S1645*^ fibroblasts shown by anti-acetylated tubulin (shown in red). IFT is detected as anti-IFT88 expression (shown in green) along the ciliary axoneme possibly accumulating at the tip. (e) The proportion of IFT88+ cells in WT fibroblasts (*n* = 94), *ALMS1* (*n* = 95), PTC124-treated (*n* = 98) and amlexanox-treated (*n* = 100) ALMS1^S1645*/S1645*^ fibroblasts. *P* < 0.05 (*), *P* < 0.01 (**), *P* < 0.001 (***). Scale bar: 10 µm. * indicates IFT88 expression.

**Fig. 8:**
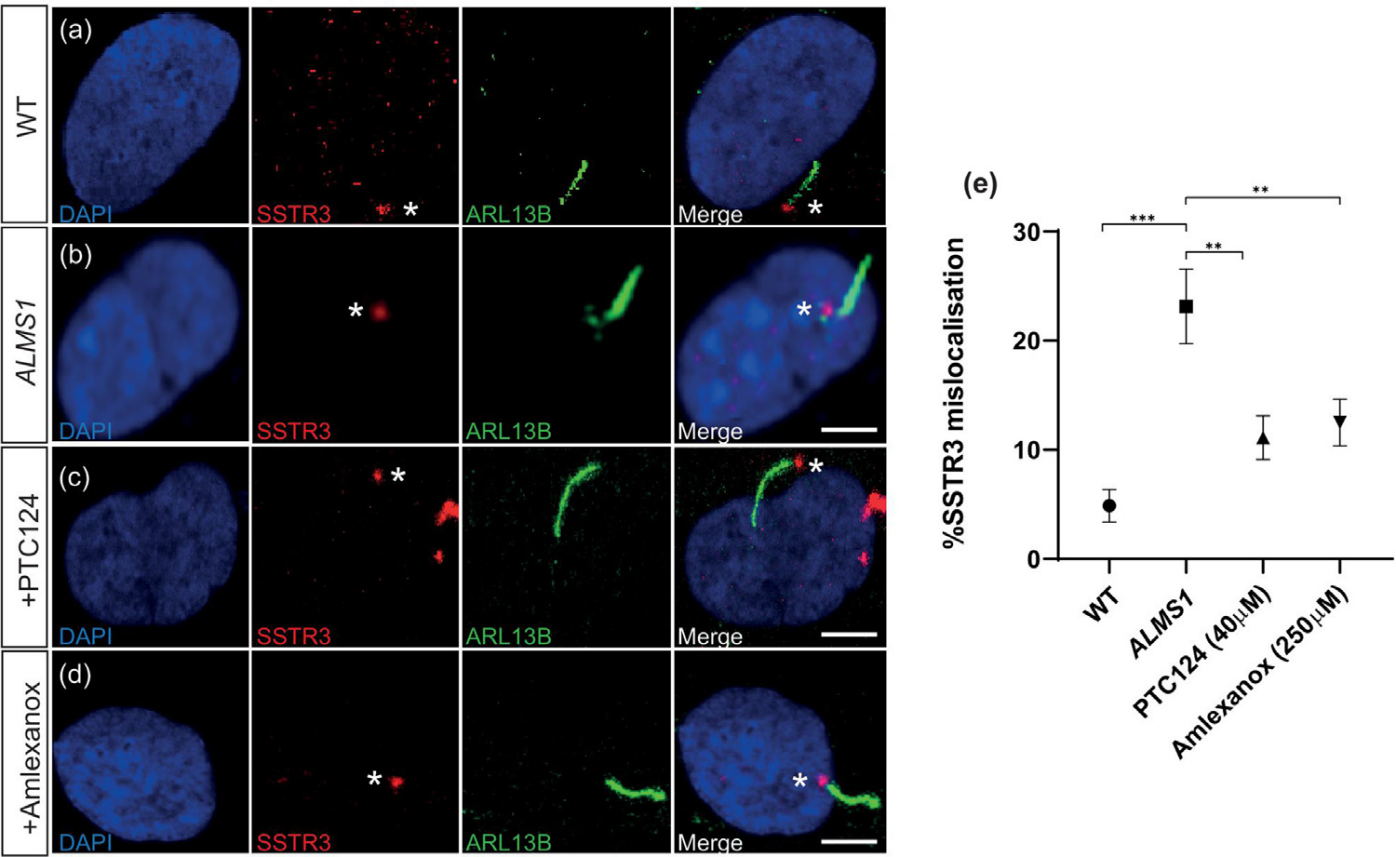
SSTR3 localisation is restored in ALMS1^S1645*/S1645*^ fibroblasts following treatment with TRIDs. (a-d) Representative immunofluorescent staining of cilia in (a) wild type (WT), (b) *ALMS1 (untreated),* (c) PTC124- and (d) amlexanox-treated ALMS1^S1645*/ S1645*^ fibroblasts shown by anti-ARL13B (shown in green) staining the ciliary membrane and anti-SSTR3 (shown in red) staining adjacent to the ciliary membrane. (e) The percentage of correct SSTR3 localisation adjacent to the ciliary membrane in WT (*n* = 108), *ALMS1* (*n* = 102), PTC124-treated (*n* = 99) and amlexanox-treated (*n* = 98) ALMS1^S1645*/ S1645*^ fibroblasts. *P* < 0.05 (*), *P* < 0.01 (**), *P* < 0.001 (***). Scale bar: 10µm. * indicates SSTR3 expression.

Similar to BBS2^Y24*/R275*^ fibroblasts, IFT88 expression was reduced to 18.95 ± 6.89% (*n* = 18/95) in untreated ALMS1^S1645*/S1645*^ fibroblasts, suggesting a significant defect of ciliary transport machinery (*P* < 0.0001, Student's *t*-test) (Fig. 7a-b). IFT88 expression was significantly restored upon treatment with PTC124 and amlexanox to 58.16 ± 4.09% (*n* = 57/98) (*P* < 0.011, Student's t-test) and 59 ± 5.03% (59/100) (*P* < 0.0078, Student's *t*-test), respectively (Fig. 7c-e). SSTR3 mislocalisation suggestive of defective ciliary protein trafficking was also observed in ALMS1^S1645*/S1645*^ fibroblasts where SSTR3 was expressed directly adjacent to the ciliary axoneme in only 76.92 ± 3.42% of cells (78/102) (*P* < 0.011, Student's t-test) (Fig. 8a-b). However, following treatment with PTC124 or amlexanox, correct SSTR3 localisation was significantly improved to 88.89 ± 1.99% (*n* = 88/99) (*P* < 0.0095, Student's t-test) and 87.76 ± 2.14% (*n* = 86/98) (*P* < 0.0031, Student's t-test), respectively (Fig. 8c-e). These data suggest a recovery of ciliary function in ALMS1^S1645*/S1645*^ fibroblasts following treatment with TRIDs.

## 4 Discussion

This is the first study to describe the correction of anatomical and functional ciliary defects in two human ciliopathy models and is an encouraging step towards the development of future therapeutics. We investigated the efficacy of two translational readthrough inducing drugs (TRIDs), PTC124 and amlexanox, as a potential treatment to restore full-length BBS2 and ALMS1 protein in BBS2^Y24*/R275*^ and ALMS1^S1645*/S1645*^ patient fibroblasts, respectively. We observed a significant restoration of between 35–40% full-length BBS2 and ALMS1 protein compared to wild type levels following treatment with either drug compound.

The *BBS2* cell model system gave rise to defective ciliogenesis, but it is still not clear to what extent human cell-specific cilia dysfunction contributes to the systemic BBS phenotype [58,59]. The impaired ciliogenesis (cilia length and ciliation) in human fibroblasts may be a cell-specific effect as observed in mouse studies [55,58,59]. In contrast, no anatomical ciliary defects were observed in *ALMS1* fibroblasts, analogous to previous reports [37,55,60]. However, through the use of IFT88 and SSTR3 localisation studies, defects in ciliary transport were recorded in both disease models, which were corrected following treatment. The loss of IFT88 expression in dermal fibroblasts has been previously reported as a readout for impaired IFT in Jeune and Mainzer-Saldino ciliopathy patients [61,62]. Here, the recovery of IFT88 to similar expression patterns along the ciliary axoneme described in healthy control fibroblasts is indicative of a return of protein trafficking within the cilium and a reliable readout of cilia function following the restoration of BBS2 and ALMS1 protein expression [62].

SSTR3 is a G protein-coupled receptor (GPCR) and its correct trafficking is important for the relay of cues from the extra-cellular environment [63]. Sstr3 mislocalisation has previously been reported in the *Bbs2^−/−^* mouse model due to a complete failure to localise to the ciliary axoneme despite high expression levels [64]. Its localisation is known to be dependent on the BBSome complex [65].

In WT dermal fibroblasts we detected SSTR3 at the basal body, directly connecting to the ciliary axoneme, although not entering the cilium. Failure to enter the cilium might reflect cell type-specific entry mechanisms. Even so, in both patient cell lines we observed subtle mislocalisation of this signal, slightly adjacent to the ciliary axoneme, which could be restored upon treatment. This mislocalisation is known to disrupt protein signalling cascades in patient cilia [19]. This is particularly relevant for the treatment of rod-cone dystrophies, which are highly prevalent in BBS and AS, as other visual GPCRs such as rhodopsin are trafficked via the BBSome across the connecting cilium to the outer segment of photoreceptor cells [66]. The correction of SSTR3 mislocalisation following restoration of BBS2 and ALMS1 expression is indicative of repaired cargo transport to the cilium and is an additional readout for cilia function.

We have used immunofluorescence analysis of cilia transport machinery and cargo as readouts of functionality, yet alternative readouts for the effect of TRIDs are possible. Changes to intracellular Ca^2+^, cAMP and insulin levels have been associated with ciliary dysfunction in *in vitro* BBS models while increased oxidative stress and loss of inflammatory cytokine action have been previously associated with impaired ciliation [67], [68], [69], [70]. Initially, these defects will need to be characterised in ciliopathy models but may act as alternative outcome measures for future studies treating ciliopathies with TRIDs.

The shared clinical phenotype of BBS and AS ciliopathies suggests related roles for ALMS1 and BBSome proteins in cilia function and protein transport [71]. ALMS1 is localised to the basal body, where filaments protruding from the basal body complex comprise the centrosome linker that maintains the pairing of the mother and daughter centrioles [37]. ALMS1 has previously been shown to have a role in the recruitment of CEP250/C-Nap1, a protein that anchors the centrosome linker and a similar structure known as the ciliary rootlet [72]. It is possible that ALMS1 is involved in tethering proteins to the basal body complex, including SSTR3. The loss of ALMS1 may result in the incorrect tethering of SSTR3 and consequent mislocalisation to the daughter centriole as the centrosome linker previously connecting the two is lost, resulting in protein trafficking defects such as those detected here. Due to the functional rather than structural cilia anomalies caused by the loss of ALMS1, further study of ALMS1^S1645*/S1645*^ fibroblasts should focus on ciliary dysfunction. Scanning electron microscopy revealed minimal structural alterations in *Alms1^−/−^* murine cochlear cilia [73]. Ultrastructural analysis of ALMS1^S1645*/S1645*^ fibroblasts may reveal structural anomalies that were not apparent from immunofluorescence analysis although this is considerably more challenging in primary rather than motile cilia [74]. In this study, immunofluorescence has revealed SSTR3 mislocalisation and loss of intraflagellar transport. Alternative techniques such as the use of semi-permeable cells that enable enhanced characterisation of the diffusion barrier between the cytosol and cilium, such as the precise visualisation of ciliary protein trafficking in and out of the axoneme, may reveal previously unknown ciliary dysfunction underlying BBS and AS [75,76]. In addition, despite the immense technical challenges in capturing primary cilia, electron microscopy techniques may enhance our understanding of structural and functional ciliary anomalies contributing to BBS and AS pathophysiology such as impaired vesicle transport from the Golgi apparatus to the cilium [74,76].

Further studies of the precise functions of each BBS protein may advance our understanding of disease aetiology and the pleiotropic nature of BBS as increasing evidence suggests BBS proteins are not functionally restricted to the primary cilium [77,78]. Non-ciliary roles for BBS proteins have been previously described, such as regulation of the actin cytoskeleton at actin-rich sites in renal medullary cells and in the murine cochlear, and localisation to the centrosome and other cell division subunits such as dynactin to facilitate mitosis [77,79]. Furthermore a subset of BBS proteins have also been found to enter the nucleus. For instance, BBS6 is transported between the nucleus and cytoplasm and modulates the sub-cellular location of other proteins [80]. In addition, BBS7 has been shown to have dynamic localisation patterns between the nucleus, centrosome and cilium while directly regulating transcription through the expression levels of RNF2, an E3-ubiquitin ligase involved in histone code and gene regulation, and its target genes [81]. Dissecting the non-ciliary versus ciliary roles of these proteins in non-ciliated cells such as lymphocytes may advance our knowledge of global BBS protein functionality and improve development of novel therapies. ALMS1 also has purported non-ciliary roles such as the regulation of cell cycle machinery and proteasome, and in the cellular DNA damage response [37]. Future studies assessing the loss of ALMS1 in these functions will provide insight into possible contributions of non-ciliary roles of ALMS1 to AS pathophysiology.

The shared functional rescue in both in both BBS2^Y24*/R275*^ and ALMS1^S1645*/S1645*^ fibroblasts is encouraging for the widespread use of TRIDs to treat a range of ciliopathies caused by different nonsense mutations in a disease- and gene-independent approach [46]. Our data confirmed the dual mechanism of action of amlexanox, in terms of NMD inhibition and nonsense suppression, in both BBS and AS models. PTC124 has traditionally not been considered to exhibit NMD inhibition activity, although an increase in mRNA transcript levels was noted in the BBS2 patient fibroblasts, but not in the AS cellular model following treatment. In a previous study testing PTC124 in choroideremia models, increased *chm* mRNA transcripts were detected in the *in vivo chm^ru848^* zebrafish, but not in the *CHM^Y42X/y^* patient fibroblasts [49].

The baseline level of mRNA is likely to be a prognostic indicator, with a minimal level that guarantees enough substrate for readthrough. Linde et.al reported that in a cohort of cystic fibrosis patients with an identical *CFTR* mutation p.(W1282*), patients with higher baseline mRNA levels demonstrated higher readthrough rates following gentamicin treatment [82]. A previous study reported no significant rescue following treatment of CHM^K258*^ fibroblasts and corresponding patient iPSC-derived RPE with PTC124, which had low baseline *CHM* transcript levels (~20%) [43]. Sarkar et al. showed highly variable *CHM* mRNA levels between patients with the same nonsense mutation [c.715C>T; p.(R239*)] ranging from 13–52.6%, but conservation in transcript levels between different tissue types from the same patient (blood, dermal fibroblasts and iPSC-derived RPE) [57]. The control/triggers of NMD are not fully understood, and variable levels of mRNA have also been documented in different tissues of the same patient [83]. It is likely that increased transcript abundance will improve readthrough efficiency, and could be used for future treatment stratification.

It has been suggested that certain TRIDs have a readthrough preference depending on the context of PTC (most commonly UGA>UAG>UAA) and the surrounding 5’ (most commonly *A*>*G*=*C*>*U*) and 3’ sequence [84]. Adenine is most preferred in the 5’ position for highest readthrough whereas cytosine is 3-6 times more effective in the 3’ position such as UGA-C in all TRID studies even though the optimal subsequent order of bases is TRID-dependent [84], [85], [86], [87]. Interestingly, the base immediately following the stop codon exerts the strongest influence on readthrough efficiency due to the interaction of mRNA with release factors to terminate translation [84,88]. Computer modelling has suggested PTC124 has a preference for UGA readthrough due to a more favourable binding energy than UAG or UAA stop codons [89]. In addition, PTC124 readthrough efficiency is similarly influenced by the context of the PTC as previously described, preferentially interacting with adenine upstream of the PTC and cytosine immediately downstream of the PTC [89]. Further studies are required to assess the effects of varying PTC contexts on amlexanox readthrough efficiency. The BBS2^Y24*/R275*^ patient mutations represent a UAG and UGA, respectively, but neither are flanked by optimal nucleotides. The ALMS1^S1645*/^^S1645*^ patient harbours two UAA stop codons and is surrounded by thymine and guanine at the 5’ and 3’ ends, respectively. As the restoration of protein was not statistically different between the drugs, nor between the two disease models, this suggests that the drugs have no predictable preference.

This study provides proof-of-concept for the clinical application of TRIDs for ciliopathies. PTC124 is currently optimised for oral administration three times a day, at a recommended dose of 10 mg/kg in the morning and at midday, and 20 mg/kg in the evening. The most commons side effects, which affect more than 5 in 100 people are vomiting, diarrhoea, nausea, headache and flatulence. No serious adverse events have been noted with over 150 patients monitored over ten years and another 180 over five years. Hence this form of delivery may be particularly beneficial for syndromic diseases with multiple disease targets as seen with ciliopathies. Bioavailability to certain tissues such as the retina may require local delivery to bypass the blood-retinal barrier through intravitreal or subretinal injection, although in most patients with existing retinal degeneration this boundary is weakened. The START formulation (0.9% Sodium chloride, 1% Tween 80, 1% powdered PTC124, and 1% carboxymethylcellulose) greatly improved the delivery of PTC124 to the mouse retina and cornea [90], however this has not yet been tested in higher order animals or patients. A recent phase II clinical trial of the safety and efficacy of PTC124 for the treatment of nonsense-mediated aniridia (NCT02647359) failed to meet the primary outcome measure of a change in maximum reading speed from baseline with both eyes (using the MNREAD Acuity Charts) [91]. Further analyses of secondary endpoints are underway, but this also highlights the requirement to ensure the correct clinical outcome metrics are selected for trial measures. This may be easier with ciliopathies due to the widespread gene expression patterns and multisystemic features.

Our data suggest that TRIDs may be a suitable therapeutic approach for nonsense mutation-associated ciliopathies. TRIDs have been largely utilised to treat diseases such as Duchenne Muscular Dystrophy with a strong genotype-phenotype correlation with the most severe phenotype associated with nonsense variants [92]. Both BBS and AS lack clear genotype-phenotype correlations, thus further *in vivo* studies are required to assess the safety and systemic efficacy of TRID treatment on a range of phenotypes associated with both ciliopathies prior to translation to clinical trials. Animal models of nonsense-associated BBS and AS such as *Bbs2-, Bbs4-, Alms1-* and *alms1*-null mice and zebrafish have been previously reported and can be utilised for future studies [93,94] The restoration of full-length proteins BBS2 and ALMS1 and the correction of both anatomical and functional ciliary defects reported in our proof-of-concept study hold immense promise for future ciliopathy research and importantly, the patients affected by these syndromes.

## Contributors

Conceptualisation: HMS, MM. Investigation: JE. Methodology: JE, HMS, MM. Resources: EF, MM. Supervision: HMS, MM. Laboratory experimental analyses: JE, HMS, MM. Statistical analyses: JE, HMS, MM. Writing – original draft: JE. Writing – review and editing: EF, HMS, MM. All authors read and approved the final draft and have had access to the raw data. Funding acquisition: MM

## Data sharing

Individual patient data are protected for confidentiality reasons. All relevant data have been presented in the manuscript. Requests for data, study protocols or any other relevant inquiries can be made to m.moosajee@ucl.ac.uk

## Funding

Wellcome Trust 205174/Z/16/Z, National Centre for the Replacement, Refinement & Reduction of Animals in Research. Deutsche Forschungsgemeinschaft SPP2127 (DFG Grant MA 6139/3-1).

## Declaration of Competing Interest

The authors report no conflict of interest

## Appendix Supplementary materials

Image, application 1

Image, application 2
