# Supplementary material for: Translational readthrough of ciliopathy genes BBS2 and ALMS1 restores protein, ciliogenesis and function in patient fibroblasts
Source: eBioMedicine. 2021 Aug 5;70:103515. doi: 10.1016/j.ebiom.2021.103515 (PMC8353411; doi:10.1016/j.ebiom.2021.103515)
Supplement: Supplementary file 1 [file mmc1.docx]

**Supplementary material**

**Study protocol**

*Fibroblast derivation and maintenance*

1. Extract a 5mm^2^ punch from the patient using local anaesthetic.
2. Place skin biopsy in 400µL derivation media (DMEM-High glucose-GlutaMAX Supplement pyruvate, 20% FBS, 0.25% collagenase 1, 0.05% DNase 1, 1% penicillin/streptomycin (P/S))
3. Incubate for 24 hours overnight at 37°C. The epidermis should detach, and the dermis will disintegrate.
4. Vortex the sample to disrupt the skin
5. Add 3.5mL derivation media (DMEM, 20% FBS, 1% P/S) to the tube containing the biopsy.
6. Mix well 2 or 3 times and plate into a T25 flask.
7. Incubate at 37°C for 3 days without disturbance.
8. Add 5mL derivation media once a healthy growth of fibroblasts is visible, or at day 7.
9. Change the media with fibroblast media (DMEM, 15% FBS, 1% P/S) every 4-5 days.
10. Passage every 7-10 days or when cells reach ~80% confluency using TrypLE ™ Express.
11. Maintain fibroblasts in DMEM, 15% FBS and 1% penicillin/streptomycin (P/S).


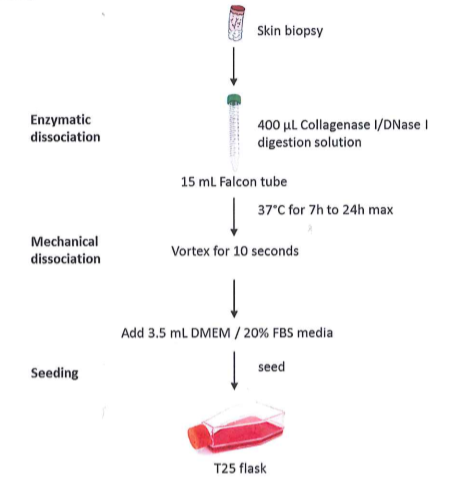


*Treatment of cells*

1. Remove serum from fibroblast media for 24 hours
2. Passage cells when ~80% confluent using TrypLE ™ Express.
3. Count 600 000 cells for seeding into one well of a 6-well plate or 300 000 cells for seeding into one well of a 12-well plate.
4. Maintain cells overnight in serum-free media for 24 hours.
5. Prepare 100µM and 500µM stock solutions of PTC124 and amlexanox respectively. Suspend drugs in 1mL PBS before adding required volume of fibroblast media.
6. Prepare working solutions of 40µM and 250µM of PTC124 and amlexanox respectively.
7. Replace cell culture media with working solutions of TRIDs. 2mL for each well of a 6-well plate, 1mL for each well of a 12-well plate and 500µL for each well of a 24-well plate.
8. Replace TRID working solutions with fresh working solution approximately 12 hours following first dose.
9. Harvest cells for downstream applications 24 hours following first dose.

*Western Blot*

1. Prepare lysis buffer on ice. Use 1mL RIPA buffer for 5x10^6^ cells or adjust volume accordingly. Add 1:100 volume of protease/phosphatase inhibitor cocktail.
2. Add appropriate volume of prepared buffer to each cell pellet, mix well and keep on ice.
3. Sonicate 2x10second pulses at 4 amplitude microns. Keep on ice before and after.
4. Incubate on ice for 10 minutes.
5. Centrifuge samples at maximum speed for 10 minutes at 4°C.
6. Collect supernatant and transfer to new tubes. Keep on ice while preparing BCA assay.
7. Set incubator at 37°C and prepare standards in duplicate according to following table

| Standard | Concentration |
| --- | --- |
| 1 | 1500µg/mL |
| 2 | 1000µg/mL |
| 3 | 750µg/mL |
| 4 | 500µg/mL |
| 5 | 250µg/mL |
| 6 | 125µg/mL |
| 7 | 50µg/mL |
| 8 | 25µg/mL |
| 9 | 5µg/mL |
| 10 | 0µg/mL |

1. Prepare BCA working reagent. 100µL solution A + 5µL solution B per well of a 96-well plate. Multiply this by total wells to calculate total volume required. All wells to be done in duplicate.
2. Add 100µL BCA working reagent and 10µL of standard/sample per well.
3. Incubate plate at 37°C for 30 minutes.
4. Leave plate to reach room temperature and read absorbance at 562nm
5. Calculate protein concentrations in Excel
   1. Average the 2 absorbance readings per sample/standard.
   2. Subtract the blank reading from the average reading for each sample/standard.
   3. Create scatterplot by graphing absorbance over concentration.
   4. Add a linear trend line and R^2^ value on the graph.
   5. Rearrange equation so that x is the subject of the equation and the unknown concentrations of the samples can be calculated.
   6. Calculate concentrations of unknown samples and divide by 1000 to convert units to µg/µL
6. Prepare running buffer = 40mL MES running buffer + 760 mL ddH_2_O
7. Prepare samples on ice
   1. Add 5µL sample buffer per sample or 10µL per duplicate (A).
   2. Add 2µL reducing agent per sample of 4µL per duplicate (B).
   3. Add calculated volume to give 30µg total protein (C).
   4. Add 20µL – (A+B+C) ddH_2_O per sample or 40µL – 2(A+B+C) ddH_2_O per duplicate.
   5. Incubate samples at 100°C for 5 minutes.
   6. Spin samples at room temperature at 13 000 x g for 5 minutes.
8. Prepare precast gel
   1. Take gel cassette out of the packet
   2. Rinse gel cassette with water and remove strip at the bottom
   3. Place one (use mock gel for second slot if only running one gel) or two gels in casket and clamp shut to prevent leakage.
   4. Place into gel tank and orientate to correct electrodes.
   5. Pour running buffer into the middle of the casket to overflow. Once buffer covers well, pour rest of the buffer until the correct level shown on tank.
   6. Gently remove the cover from the wells and flush to remove bubbles and align.
9. Load 3µL of pre-stained protein standards as ladder
10. Load 20µL of sample in appropriate well
11. Load 5µL sample buffer into any empty wells
12. Run the gel at 85-100V for approximately 1.5 hours and watch the ladder to determine the best length.
13. Prepare the transfer buffer = 20mL transfer buffer + 50mL 100% methanol and 330mL ddH_2_O.
14. Prepare dipping trays – one with transfer buffer and the other with methanol
15. Cut two pieces of blot paper and one piece of PVDF membrane. Place the blot paper in the transfer buffer tray.
16. Soak the PVDF membrane in methanol for 30 seconds then transfer to the transfer buffer.
17. Remove the gel from the gel tank, rinse with water and crack open the cassette with the tool provided – remove half of the cassette and leave the gel on the other.
18. Trim around the gel.
19. Assemble the transfer sandwich on the semi-dry transfer machine. Place the blot paper, followed by the membrane. Roll out the bubbles. Place the gel with the correct orientation on the membrane and roll out bubbles. Place the second soaked blot paper on top.
20. Make sure the surrounding area is completely dry.
21. Calculate the correct amount of current to transfer through the formula 0.8 x length x width of the sandwich. Run at this current for approximately 1 hour.
22. Following transfer, block the membrane at room temperature in 5% milk 0.1% PBS/Tween solution.
23. Based on the manufacturer’s recommendations, prepare the primary antibody solution in the 5% milk 0.1% PBS/T solution and incubate overnight at 4°C. For BBS2, this was 1:800 and for β-actin this was 1:5000.
24. Wash the membrane 5 times for 5 minutes each at room temperature.
25. Incubate the membrane with the appropriate secondary antibody dilution for one hour at room temperature. Secondary antibodies were applied at 1:5000.
26. Wash the membrane again 5 times for 5 minutes each at room temperature.
27. Prepare a 1:1 mix of the ECL prime detection substrate solution. Incubate membrane in this solution following the washes for 5 minutes at room temperature.
28. Set appropriate parameters and image using ChemiDoc and ImageLab software.

*RT-qPCR*

1. Extract RNA from cell pellets using QIAGEN RNeasy MicroKit according to the manufacturers’ recommendations.
2. Quantify extracted RNA.
3. Synthesize up to 1µg of cDNA using First Strand Synthesis Kit III according to manufacturers’ instructions and adjust concentration to 5ng/µL.
4. Prepare each well as follows: 10µL Sybr Master Mix (QIAGEN), 2µL cDNA, 2µL primer pair, 6µL ddH_2_O. Prepare each sample in triplicate with *GAPDH, ACTB* and *G6PD* used as housekeeping reference genes.
5. Run the following thermocycling protocol:
6. Transcript levels were normalised to wild-type fibroblasts and quantified using the comparative C_t_ method. This method first determines the difference between C_t_ values (ΔC_t_) of each gene of interest and reference gene for each experimental sample. This calculation was performed individually for each reference gene and the average ΔC_t_ value was calculated and used for downstream analysis. The difference in the ΔC_t_ values between experimental and control samples (ΔΔC_t_) is calculated. The fold change in expression of the gene of interest between the two samples is equal to 2^-ΔΔCt^.
7. Generate graphs using GraphPad Prism and perform unpaired t-tests between experimental groups to determine significance.

*Immunocytochemistry*

1. Prior to treatment, cells were plated on sterilised cover slips and plated as per above.
2. Cells were treated as described above.
3. Following treatment, wash cells with PBS.
4. Fix with ice-cold methanol for 10 minutes at room temperature.
5. Wash cells with PBS.
6. Permeabilise cells for 20 minutes at room temperature with 0.3% PBS/Triton-X with slight agitation.
7. Wash cells with PBS 5 minutes at room temperature.
8. Block cells for 1 hour at room temperature with 10% normal goat serum (NGS) 0.3% PBS/Triton-X.
9. Prepare primary antibody solutions for overnight incubation at 4°C in 10% NGS, 0.3% PBS/Triton-X. The dilutions were prepared as follows:

| **Antibody** | **Catalogue number** | **RRID** | **Host (dilution)** | **Application** |
| --- | --- | --- | --- | --- |
| Anti-SSTR3 | Invitrogen, CA, USA, PA3-110 | AB_2302616 | Rabbit (1:1000) | Immunocytochemistry (ICC) |
| Anti-IFT88 |  |  | Rabbit (1:800) | ICC |
| Anti-ARL13B | Antibodies Incorporated, CA, USA, N295B/66 | AB_2750771 | Mouse (1:800) | ICC |
| Anti-ARL13B | Proteintech, IL, USA, 17711-1-AP | AB_2060867 | Rabbit (1:500) | ICC |
| Anti-acetylated tubulin | Sigma-Aldrich, MO, USA, T6793 | AB_477585 | Mouse (1:1000) | ICC |
| Anti-ALMS1 | Abcam, UK, ab84892 | AB_1859885 | Rabbit (1:400) | ICC |

1. Wash cells 3x10minute washes at room temperature with PBS.
2. Incubate cells with secondary antibodies room temperature for 1 hour. Solutions prepared in 10% NGS, 0.3% PBS/Triton-X. The dilutions were prepared as follows:

| **Secondary antibodies used in immunocytochemistry and western blotting** | | | | |
| --- | --- | --- | --- | --- |
| **Antibody** | **Catalogue number** | **RRID** | **Host (dilution)** | **Application** |
| Goat anti-Mouse IgG (H+L) Cross-Adsorbed Secondary Antibody, Alexa Fluor 647 | ThermoFisher Scientific, MA, USA, A21235 | AB_141693 | Goat (1:1000) | ICC |
| Goat anti-Mouse IgG (H+L) Cross-Adsorbed Secondary Antibody, Alexa Fluor 488 | ThermoFisher Scientific, MA, USA, A10011 | AB_2534069 | Goat (1:1000) | ICC |
| Goat anti-Rabbit IgG (H+L) Highly Cross-Adsorbed Secondary Antibody, Alexa Fluor 488 | ThermoFisher Scientific, MA, USA, A11034 | AB_2576217 | Goat (1:1000) | ICC |
| Goat anti-Rabbit IgG (H+L) Highly Cross-Adsorbed Secondary Antibody, Alexa Fluor 647 | ThermoFisher Scientific, MA, USA, A21245 | AB_2535813 | Goat (1:1000) | ICC |

1. Wash cells 3x5minute washes at room temperature with PBS.
2. Mount coverslips onto slides using Diamond AntiFade mounting media with DAPI.
3. Image slides using ZEISS LSM710.
4. Count cilia using cell counter on ImageJ by counting cilia detected by anti-ARL13B and anti-acetylated tubulin colocalization.
5. By identifying cilia by anti-ARL13B and anti-acetylated tubulin colocalization, measure cilia length using curved line function on ImageJ. Measure in triplicate to ensure accuracy.
6. Identify SSTR3 or IFT88 expression with cilia markers ARL13B or acetylated tubulin using ImageJ to assess localisation.
