# Supplementary material for: Translational readthrough of ciliopathy genes BBS2 and ALMS1 restores protein, ciliogenesis and function in patient fibroblasts
Source: eBioMedicine. 2021 Aug 5;70:103515. doi: 10.1016/j.ebiom.2021.103515 (PMC8353411; doi:10.1016/j.ebiom.2021.103515)
Supplement: Supplementary file 2 [file mmc2.docx]

| **Supplementary Table 1**  **Primary antibodies used in immunocytochemistry and western blotting** | | | | |  |
| --- | --- | --- | --- | --- | --- |
| **Antibody** | **Catalogue number** | **RRID** | **Host (dilution)** | **Application** | **Reference** |
| Anti-SSTR3 | Invitrogen, CA, USA, PA3-110 | AB_2302616 | Rabbit (1:1000) | Immunocytochemistry (ICC) | [57] |
| Anti-IFT88 | Proteintech, IL, USA, 13967-1-AP | AB_2121979 | Rabbit (1:800) | ICC | [58] |
| Anti-ARL13B | Antibodies Incorporated,CA, USA, N295B/66 | AB_2750771 | Mouse (1:800) | ICC | [59] |
| Anti-ARL13B | Proteintech, IL, USA, 17711-1-AP | AB_2060867 | Rabbit (1:500) | ICC | [60] |
| Anti-acetylated tubulin | Sigma-Aldrich, MO, USA, T6793 | AB_477585 | Mouse (1:1000) | ICC | [49] |
| Anti-ALMS1 | Abcam, UK, ab84892 | AB_1859885 | Rabbit (1:400) | ICC | [55] |
| Anti-BBS2 | ProteinTech, IL, USA 11188-2-AP | AB_2064971 | Rabbit (1:800) | Western Blot (WB) | [55] |
| Anti-β-actin | Sigma-Aldrich, MO, USA A2228 | AB_476697 | Mouse (1:5000) | WB | [49] |
| \| **Secondary antibodies used in immunocytochemistry and western blotting** \| \| \| \| \| \| --- \| --- \| --- \| --- \| --- \| \| **Antibody** \| **Catalogue number** \| **RRID** \| **Host (dilution)** \| **Application** \| \| Goat anti-Mouse IgG (H+L) Cross-Adsorbed Secondary Antibody, Alexa Fluor 647 \| ThermoFisher Scientific, MA, USA, A21235 \| AB_141693 \| Goat (1:1000) \| ICC \| \| Goat anti-Mouse IgG (H+L) Cross-Adsorbed Secondary Antibody, Alexa Fluor 488 \| ThermoFisher Scientific, MA, USA, A10011 \| AB_2534069 \| Goat (1:1000) \| ICC \| \| Goat anti-Rabbit IgG (H+L) Highly Cross-Adsorbed Secondary Antibody, Alexa Fluor 488 \| ThermoFisher Scientific, MA, USA, A11034 \| AB_2576217 \| Goat (1:1000) \| ICC \| \| Goat anti-Rabbit IgG (H+L) Highly Cross-Adsorbed Secondary Antibody, Alexa Fluor 647 \| ThermoFisher Scientific, MA, USA, A21245 \| AB_2535813 \| Goat (1:1000) \| ICC \| \| Polyclonal Goat Anti- Mouse Immunoglobulins antibody \| Dako, Denmark, P0447 \| AB_2617137 \| Goat(1:10 000) \| WB \| \| Polyclonal Goat Anti- Rabbit Immunoglobulins antibody \| Dako, Denmark, P0448 \| AB_2617138 \| Goat(1:10 000) \| WB \| | | | | |  |

[57] Walker TL, et al. Prominin-1 allows prospective isolation of neural stem cells from the adult murine hippocampus. J Neurosci 2013;33(7):3010–24.

[58] Shaheen R, et al. Bi-allelic mutations in FAM149B1 cause abnormal primary cilium and a range of ciliopathy phenotypes in humans. Am J Hum Genet 2019;104(4):731–7.

[59] Hua K, Ferland RJ. Fixation methods can differentially affect ciliary protein immunolabeling. Cilia 2017;6:5.

[60] Capowski EE, et al. Reproducibility and staging of 3D human retinal organoids across multiple pluripotent stem cell lines. Development 2019;146(1).

| **Table 2** | |
| --- | --- |
| **RT-qPCR primer sequences** | |
| *GAPDH* forward | 5′ - CTTTTGCGTCGCCAG - 3’ |
| *GAPDH* reverse | 5′ - TTGATGGCAACAATATCCAC - 3’ |
| *ACTB* forward | 5’ - TTCTACAATGAGCTGCGTG - 3’ |
| *ACTB* reverse | 5’ - GGGGTGTTGAAGGTCTCAAA - 3’ |
| *G6PD* forward | 5’ - GAAGAGCTTTTCCAGGGCGA - 3’ |
| *G6PD* reverse | 5’ - TGAAGGTGTTTTCGGGCAGA - 3’ |
| *BBS2* forward | 5’ - GGTCGTCTGCGGGTTGGAAA - 3’ |
| *BBS2* reverse | 5’ - GCCAGGAACTTCATGACCTGTA - 3’ |
| *ALMS1* forward | 5’ - AAGCTACGCAAAGCTCCTGT - 3’ |
| *ALMS1* reverse | 5’ - TAAAGGCTGGCCATCTGTGC - 3’ |

Captions for each supplementary file

1. Step-by-step study protocol


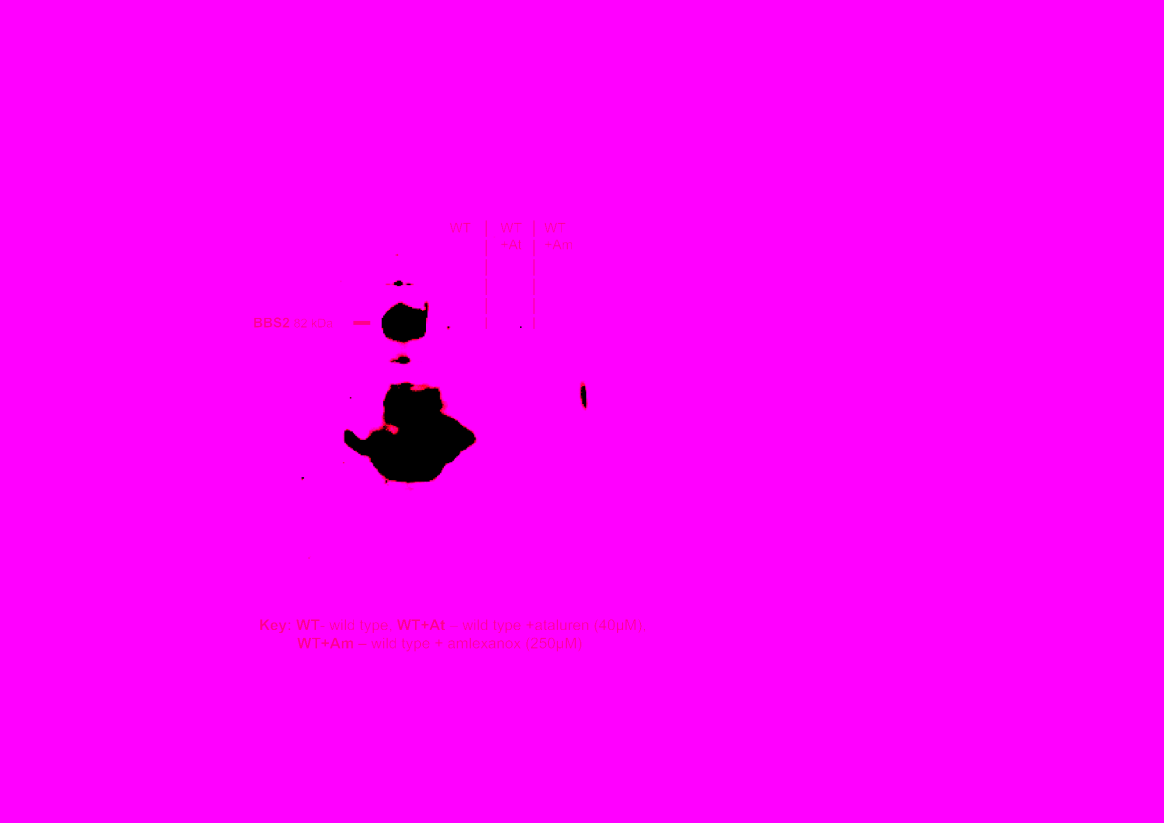


1. Complete western blot (BBS2) – Figure 1b


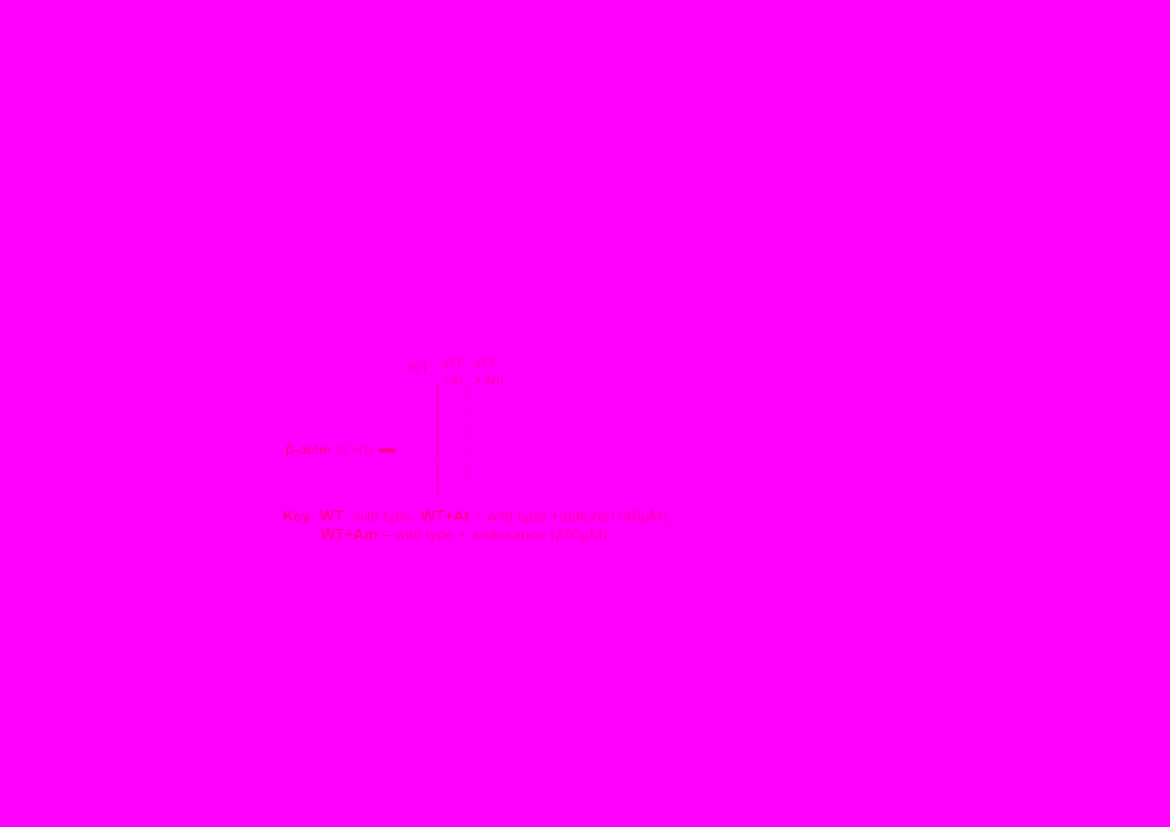


1. Complete western blot (β-actin) – Figure 1b


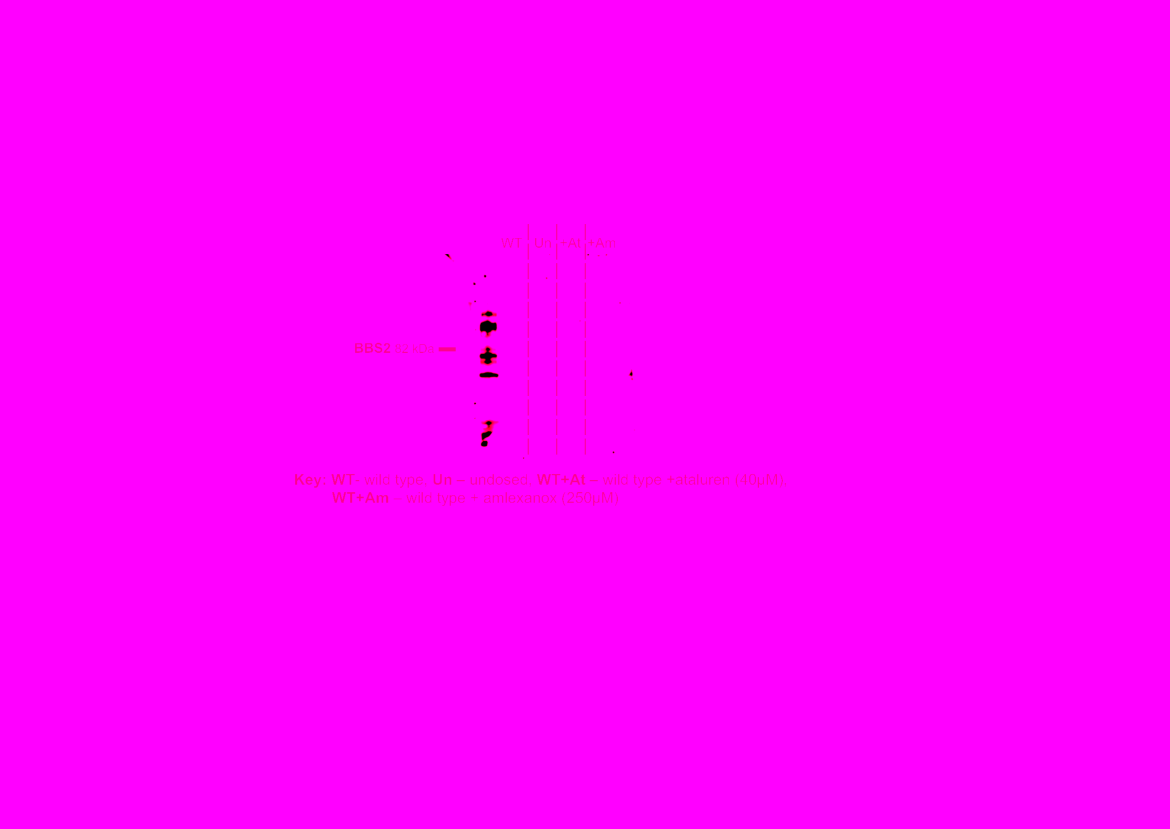


1. Complete western blot (BBS2) – Figure 1c


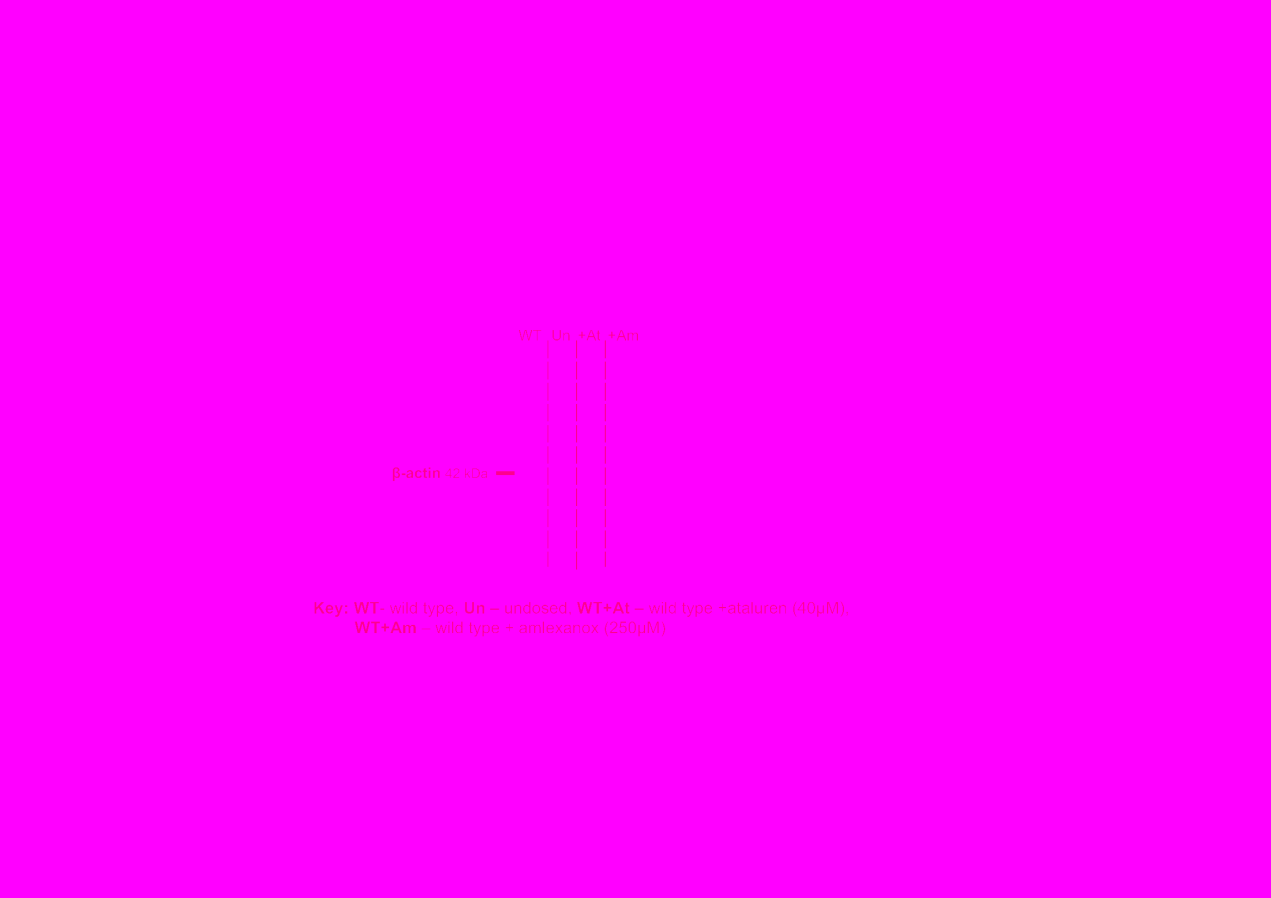


1. Complete western blot (β-actin) – Figure 1c
